# TTCA: an R package for the identification of differentially expressed genes in time course microarray data

**DOI:** 10.1186/s12859-016-1440-8

**Published:** 2017-01-14

**Authors:** Marco Albrecht, Damian Stichel, Benedikt Müller, Ruth Merkle, Carsten Sticht, Norbert Gretz, Ursula Klingmüller, Kai Breuhahn, Franziska Matthäus

**Affiliations:** 1 Complex Biological Systems Group (BIOMS/IWR), Heidelberg, Im Neuenheimer Feld 294, Heidelberg, 69120 Germany; 2 Systems Biology Group, Université du Luxembourg, 7, avenue du Swing, Belvaux, L-4367 Luxembourg; 3 CCU Neuropathology Group, German Cancer Research Center (DKFZ), Im Neuenheimer Feld 221, Heidelberg, 69120 Germany; 4 Institute of Pathology, Heidelberg University Hospital, Im Neuenheimer Feld 672, Heidelberg, 69120 Germany; 5 German Cancer Research Center (DKFZ), Im Neuenheimer Feld 280, Heidelberg, 69120 Germany; 6 Translational Lung Research Center (TLRC), Member of the German Center for Lung Research (DZL), Im Neuenheimer Feld 430, Heidelberg, 69120 Germany; 7 Medical Research Center, Medical Faculty Mannheim, University of Heidelberg, Theodor-Kutzer-Ufer 1-3, Mannheim, 68167 Germany; 8 Frankfurt Institute for Advanced Studies (FIAS), Goethe University Frankfurt, Ruth-Moufang-Straße 1, Frankfurt am Main, 60438 Germany

**Keywords:** Differential expression, Time series, EGF, Stimulation experiments, Gene ontology, Gene set analysis

## Abstract

**Background:**

The analysis of microarray time series promises a deeper insight into the dynamics of the cellular response following stimulation. A common observation in this type of data is that some genes respond with quick, transient dynamics, while other genes change their expression slowly over time. The existing methods for detecting significant expression dynamics often fail when the expression dynamics show a large heterogeneity. Moreover, these methods often cannot cope with irregular and sparse measurements.

**Results:**

The method proposed here is specifically designed for the analysis of perturbation responses. It combines different scores to capture fast and transient dynamics as well as slow expression changes, and performs well in the presence of low replicate numbers and irregular sampling times. The results are given in the form of tables including links to figures showing the expression dynamics of the respective transcript. These allow to quickly recognise the relevance of detection, to identify possible false positives and to discriminate early and late changes in gene expression. An extension of the method allows the analysis of the expression dynamics of functional groups of genes, providing a quick overview of the cellular response. The performance of this package was tested on microarray data derived from lung cancer cells stimulated with epidermal growth factor (EGF).

**Conclusion:**

Here we describe a new, efficient method for the analysis of sparse and heterogeneous time course data with high detection sensitivity and transparency. It is implemented as R package TTCA (transcript time course analysis) and can be installed from the Comprehensive R Archive Network, CRAN. The source code is provided with the Additional file 1.

**Electronic supplementary material:**

The online version of this article (doi:10.1186/s12859-016-1440-8) contains supplementary material, which is available to authorized users.

## Background

**Fig. 1:**
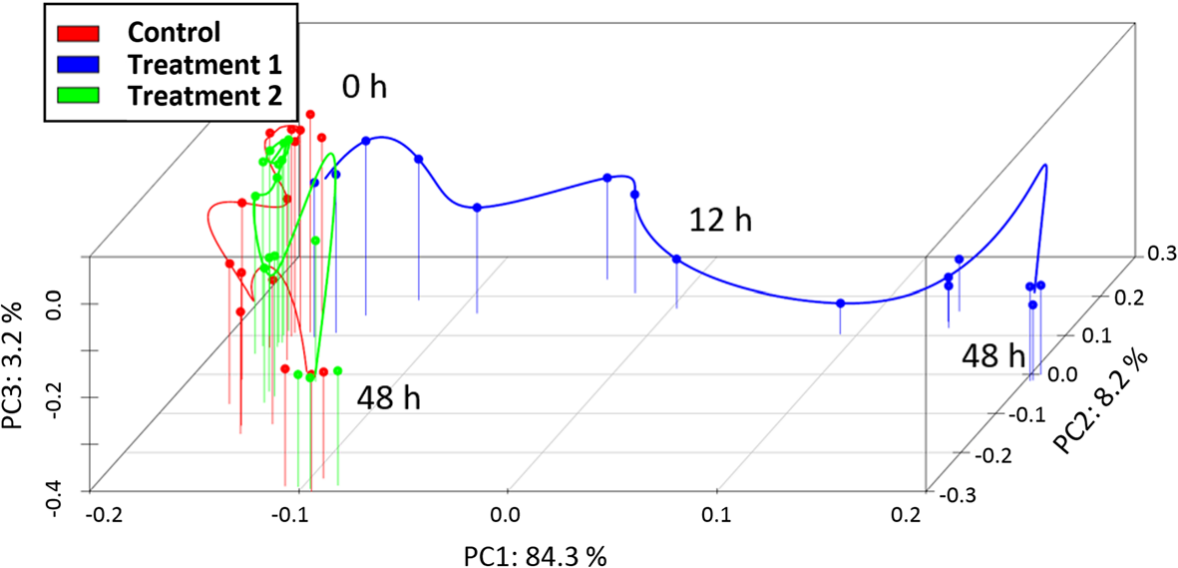
Trajectory of the transcriptomes: Axes represent principal components explaining 95.7% of the variability in the data. Measurement points represent the entire transcriptome under three different stimulation experiments projected onto the first three principal components. For early time periods, all three transcriptomes correlate very well with each other. Over time, the transcriptomes develop stimulus dependent. Stimulus 1 leads to a strong change in the transcriptome, while stimulus 2 has a much smaller effect. Possible outliers are measurement points that show a large distance from the trajectory or from related replicates

Time course microarray experiments are frequently conducted to study the dynamics of gene expression at several consecutive time points. Associated data sets often require own custom-made analysis strategies, and cannot been adequately exploited with standard methods which were established to compare groups. The variability of the dynamics, spanning from fast and transient to slower, long-lasting changes, is a challenge for the analysis of time series microarray data. In perturbation experiments, sampling frequency is often adapted to reflect the expected changes in gene expression. This kind of experimental design leads to irregularly sampled data sets. Irregular time sampling may also arise when time points are chosen to be omitted after quality control, for instance when the respective arrays represent outliers with respect to the global trajectory resulting from principal component analysis (PCA) as shown in Fig. 1. If replicates are considered, their number may also vary due to the experimental design or quality issues. Often time course-data provide only one replicate per time point.

The first methods applied on time course microarrays including SAM [1], ANOVA [2] and Limma [3] where extensions of methods for contrasts between states and do not include the order of time points into the analysis [4].

EDGE was one of the first methods taking the time sequence into account [5, 6]. EDGE involves a fit of natural cubic splines to gene expression profiles, and a bootstrap approach providing a reference distribution. MaSigPro (Microarray Significant Profiles) operates in a similar manner [7]. Sohn et al. have modified EDGE by using a permutation-approach and controlling the family wise error rate [8]. Later, they applied the FWER as a significance threshold and made the method more robust using quantile regression [9]. These methods have three drawbacks when used to analyse sparse data containing sharp transient expression changes. First, the information of the time course measurements is underestimated. Biologically meaningful peaks might be overlooked when the related measurement points are rejected as outliers. Second, the information of the permuted reference time course is overestimated. The permutation of the measurement points within the time sequence is often used to produce reference data of the same distribution, but without the original ordered pattern of dynamic changes. This estimation of the error rate can fail in sparse data sets when the expression dynamics exhibit a sharp peak. Here, permutation of the time points merely shifts but does not wipe out the peak. With this method, the signal-to-noise ratio of genes displaying fast variations in expression can be underestimated and related genes are erroneously removed from analysis. The third problem is that a large number of computationally expensive permutations is required, to avoid granularity in the resulting ranking [4]. Granularity refers in this case to hundreds of genes with exactly the same p-value. Repeated application of the method may shift a gene to another *p*-value cluster, which impedes reproducibility of the results.

An alternative method using multivariate empirical Bayes statistics and one-sample Hotelling *T* ^2^ statistics is implemented in the R package timecourse [10]. This package does not provide a significance threshold and requires a minimum number of replicates. Also, BETR (Bayesian Estimation of Temporal Regulation) [11], which uses random-effects models and considers co-expression, relies on time point replicates. Network-based methods combine cluster analysis with detection of differential expression and focus also on co-expression [12, 13]. But co-expression is a very strict assumption for the extraction of differentially expressed genes from time course data. In tightly regulated and dynamic gene regulatory networks, it seems to be very unlikely that cells do not regulate their genes at any of the sampled time points. Some of the target genes could have a negative feedback loop and could block their own expression, which could explain fast transient dynamic changes, while other target genes could have a positive feedback loop and therefore maintain gene expression longer. Additional regulation could happen after a longer time or very fast without protein translation, i.e. with functional large non-coding RNAs [14]. Longitudinal co-expression might overlook target genes that are affected by the stimulus, but which are additionally regulated by other dynamic mechanisms. Moreover, the longer the sampled time period is, the higher is the risk that initially unaffected genes show co-expression behaviour due to completely different mechanisms without relation to the stimulus. The risk is higher to detect false positive target genes.

Methods based on Gaussian processes select differentially expressed genes from one channel experiments [15] and from two channel experiments [16], implemented in the R package gprege. However, the implemented Gaussian processes suffer from massive computational cost and the required time point replication. An alternative for two channel experiments is BATS (Bayesian Analysis of Time Series) [17, 18].

Another class of time course methods is based on principal component analysis (PCA) [19]. Inspired by a trend in the data analysis to fit the *true underlying functions* [20, 21], methods based on functional PCA (FPCA) were developed [22, 23]. The most recent method [23] can handle single replicated time course data, predict individual dynamics with PACE (Principal Component Analysis through Conditional Expectation) [24] and yields reasonable results for moderately slow expression dynamics. This method was successfully applied to clinical data derived from immune response studies [25]. For the data set considered in our study, involving perturbation experiments on cell cultures with fast expression changes, this method did not perform reliably. In particular, we observed counterintuitive differences between our original data and the original data being displayed by this method after preliminary transformation by PACE. First, the method transforms flat gene profiles into profiles exhibiting strong temporal changes, shown in Additional file 2: Figure S1A. Second, the transformed trajectories are too stiff to follow sharp peak behaviour like in Additional file 2: Figure S1B. This happens before the actual time course analysis method is applied.

Finally, even simple methods can yield good results for sparse data, for instance by computing distances or the area between curves [26, 27]. Also, a sliding window, capturing a small subset of consecutive measurement points, was discussed, but cannot be applied to non-equidistant measurements [4].

To sum up, most existing methods cannot reliably analyse sparse and irregularly sampled time course gene expression data sets. Further details and a method comparison are provided in the Additional files. A method overview is given in Additional file 2: Table S1.

## Method TTCA

The method TTCA (transcript time course analysis) includes different scores to identify genes showing differential expression dynamics of various kinds.

The *dynamics score* $\mathcal {D}_{i}$ captures slow gene expression dynamics, the *peak score* $\mathcal {P}_{i}$ selects fast transient expression changes, the *integral score* $\mathcal {I}_{i}$ accounts for absolute changes in mRNA production level in different time periods, and a *relevance score* $\mathcal {R}_{i}$ provides information on existing references in the literature. A further option allows for gene ontology groups to be processed in a similar manner as individual genes. Additionally, the *minimum overlap score* *Θ*_*i*_ is computed to identify gene ontology groups with maximal separation of the group specific expression bandwidths between two conditions. Significance threshold and effect size are calculated for each score and the *consensus score* $\mathcal {C}_{i}$ combines the different scores for a final ranking.

For the detection of differential gene expression based on two channel microarray data, we recommend to create a constant gene expression profile as control profile. This control profile might start with the expression value of the first time point, or could be set to the average expression value of the experimentally derived gene expression profile.

The gene expression level is based on an assembled set of detected probes of 25 bp length. In this article, we focus on the expression dynamics of genes, however, those probe level signals can also be mapped to related transcripts or other longer oligonucleotides. These can equally be analysed with TTCA.

In the following section, preprocessing for microarray time series data is addressed. Next, all relevant scores and components of the proposed method are explained briefly.

### Pre-processing of microarray time course data

Microarray data are usually afflicted by batch effects, i.e. unwanted variability in the samples arising from their experimental, technical and digital processing history. Batch effects can be introduced when samples are processed on different hybridisation batches (maximum 6-12 samples at once), or when a subset of the samples experienced slightly different experimental conditions (time of the day, new media, etc.). Many batch effects can be technically detected and can be removed if enough replicates are available. Microarray time course data sets are frequently sparse and the number of replicates per time point is low. In such data it is impossible to detect batch-effects [28]. Moreover, the frequently used quantile-normalisation, implemented in RMA [29], is based on the assumption that the majority of the genes shows a constant expression level. However, for time series experiments this might not be the case. Especially, cancer cells are known to have a high variability in their gene expression profiles [30]. Perturbation experiments might induce secondary gene responses that eventually result in considerable expression dynamics for a broad range of genes. It has been shown that thousands of genes can change their expression over time after stimulation [6]. Instead of using multi-array normalisation methods like RMA for time course analyses, we recommend to use within-array normalisation methods which process each array separately, independent of arrays taken at other time points. In particular, we recommend individual array standardisation with SCAN [31], which is robust against GC-content bias and some batch effects.

### Dynamics score

We define a *dynamics score* in three steps based on the method EDGE [6] and its extension using quantile-regression [9].

**Fig. 2:**
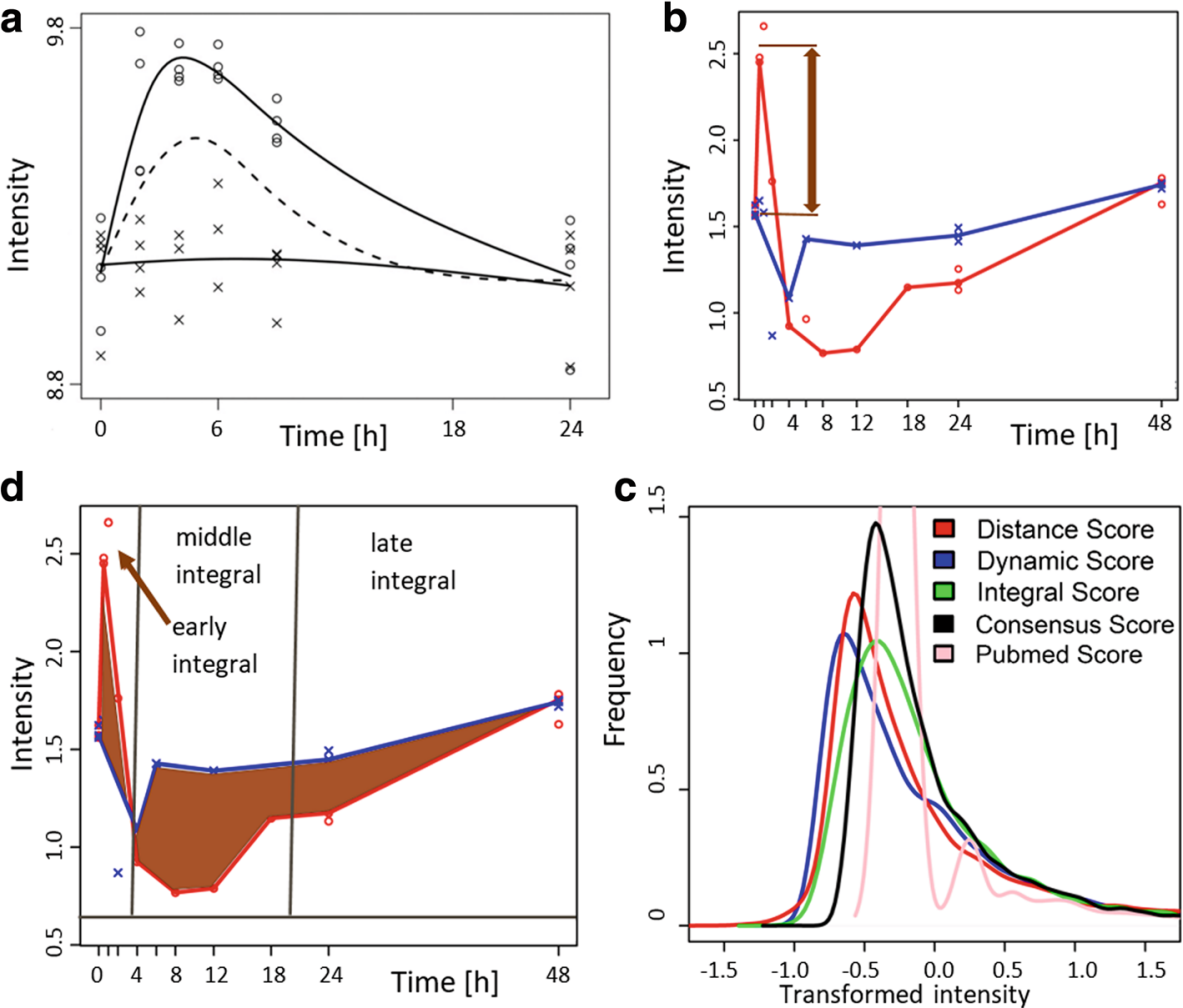
Score characteristics: **a**) *Dynamics score*. The alternative hypothesis is represented by the *solid line*. The *dashed line* represent the null hypothesis (Picture source [6]). **b**) *Peak score*. Is based on the largest distance (*arrow*) between measurement points for two different stimuli. The *solid line* represents the fit achieved via quantile regression with Eq. (1). **c**) *Integral score*. The area between two dynamics indicates the absolute mRNA production change. This value can be computed for different time intervals. **d**) Different score distributions after z-transformation and the merged *consensus score* distribution

The null hypothesis *H*_0_ is that the stimulus does not significantly alter the expression level of gene *i*. Thus, the measurements of the respective conditions (i.e. treatment vs. control) are derived from the same expression pattern and can be combined for a single function fit. In Fig. 2 a, the null hypothesis is represented by the fit to all measurement points without distinction between the conditions (dashed line). The alternative hypothesis H_1_ is that the measurements are derived from different expression patterns, and that the two conditions have to be treated separately. Hence, the data is split into the two conditions, and each time course is fitted to an individual function (see the solid lines in Fig. 2 a). The sum of the residuals of the two individual function fits should be smaller than the sum of the residuals of the single function fit to fulfil the alternative hypothesis H_1_.

The fit is based on quantile regression [32]. The fitted function *g*(*t*) and the residuals *r*_*ij*_ are obtained by minimising

1 $$ \sum_{j=1}^{n} \rho_{0.5}(y_{j}-g(t_{j}))- \underbrace{\lambda \int |g^{\prime\prime}(t)|dt}_{ \text{smoothes\ the\ function}}.   $$

The quantile regression algorithm is symbolised by *ρ*_0.5_, and implemented in the R-package Quantreg [33] in function rqss(). The index 0.5 indicates the use of the median to provide the most robust curve fit. The continuous function *g* is fitted to the measurements *y*_*j*_,*j*∈{1,…,*n*} taken at time points *t*_*j*_,*j*∈{1,…,*n*} with *n* measurements in total. The first term of Eq. (1) represents the absolute, not the quadratic distance between the measurements *y*_*j*_ and the function *g*(*t*_*j*_). Microarrays are inflicted with a certain proportion of outliers [9]. If these outliers are weighted quadratically by least-square approaches, as most methods do, a Gaussian distributed error model is assumed. However, a Gaussian error model is not a good choice for the characteristics of frequent outliers, as this approach biases the fit stronger than the absolute distance. The second term of Eq. (1) penalises the absolute number of directional changes in the gene expression dynamics to avoid over-fitting. The penalisation term is weighted by the scaling factor *λ*. We estimated *λ*=0.6 for SCAN-processed data with the help of real-time PCR profiles from genes that are known to be differentially expressed after the stimulation. The obtained residual-vectors *R*_*i*_ are modified by weighting vectors *Ω*. These weights account for the uneven experimental design in the following way: First, each time point should have the same weight independent from the number of replicates. Second, more values in one condition than in the other result in higher residuals without a better fit. TTCA balances the uneven design. Third, to reduce the unwanted bias by this vector, the sum of all vector elements of the weighting vector is forced to the same value. The scalar product of the residual and weighting vector yields a scalar value for each gene.

The *dynamics score* *D*_*i*_ is then defined by

$$\mathcal{D}_{i} := \frac{\mathrm{H}_{0}}{\mathrm{H}_{1}} = \frac{< {\Omega}^{\text{H}_{0}}, R_{i}^{\text{H}_{0}} >}{< {\Omega}^{\text{stim}},R_{i}^{\text{stim}}> + < {\Omega}^{\text{ctrl}}, {R_{i}^{\text{ctrl}}} >}. $$

The relation H_0_/H_1_ quantifies how much worse the null-hypothesis fits in comparison to the alternative hypothesis and is easy to interpret.

### Peak score

Perturbation experiments may invoke fast and transient peak dynamics in a gene subset, where the peak might be captured by only a small number of measurements. In this case, peaks, although biologically meaningful, may be overlooked by microarray analysis methods. To account for this, we introduce the *peak score*. Let *T*={*t*_1_,…,*t*_*n*_} denote the set of the measurement time points. For each time-point *t*∈*T*, we define $\mathrm {F}_{it}^{\text {stim}}$ and $\mathrm {F}_{it}^{\text {ctrl}}$ as the averages of all replicates for the stimulated and control conditions, respectively. The *peak score* is then given by

$$\mathcal{P}_{i} :=\max_{t \in T} \left|\mathrm{F}_{it}^{\text{stim}}-\mathrm{F}_{it}^{\text{ctrl}}\right|. $$

The success of this approach has been pointed out by Di Camillo et al. [26]. To test whether differences between the expression profiles are significant, we use the robust 0.95 quantile of all available standard deviations, for a minimum of 1000 genes and multiple replicated measurement points as a noise-threshold. A gene *i* is considered as significant, if $\mathcal {P}_{i}$ is more than twice the noise-threshold (see Fig. 2 b). To account for a possible correlation between the standard deviation and mean of gene expression, TTCA sorts the genes with respect to their mean values and divides them into a minimum of 8 groups, each containing at least 1000 genes. The noise-threshold is then computed separately for each group. TTCA can either use replicated time points to provide a noise threshold or the distribution of the score values to provide a significance threshold. Replicates are not required but can be used. If less than 4 measurement points are replicated, the program will provide only a ranking and the significance will be calculated as in the other scores as described below.

### Instability score

Some genes, found highly significant in the previous scores, exhibit an extreme variance between replicates. If the median of the standard deviation of replicated measurements of gene *i* is two-fold larger than the gene group noise threshold, these genes are classified as unstable. The *instability score* is binary and appears in the results table together with a relative effect size, explained below. TRUE indicates instable genes that are likely false positives, and FALSE indicates genes with acceptable variance between replicates. For an example see the gene SNORA11 in Table 1 and Fig. 4.

**Table 1:** Compendious result table. The instability of SNORA11 is confirmed and the effect size is high, which indicates a false positive result. The plotted SNORA11 profile in Fig. 4 confirms this suspicion. The effect size of the peak score covers up to 26% of the detection range

Consensus rank	Gene name	Consensus score	Consensus score *p*-value	PubMed	Instability score	Effect size of peak score
1	CTGF	1.00	3.57E-05	73	0.009	0.26
2	EGR1	0.91	7.25E-05	101	0.006	0.23
3	SNORA11	0.62	8.18E-04	0	0.038	0.26
4	PTGS2	0.59	0.001	804	0.009	0.10
5	JUN	0.58	0.001	6789	0.005	0.11
6	GLIPR1	0.57	0.001	0	0.006	0.13
7	FOS	0.55	0.002	920	0.002	0.14
8	AREG	0.53	0.002	549	0.006	0.10
13	MIR4320	0.44	0.005	0	0.016	0.15
15	F3	0.44	0.006	65	0.011	0.10
19	IL8	0.41	0.007	43	0.018	0.13
20	EGR2	0.41	0.008	7	0.005	0.12
21	PCNA	0.40	0.009	583	0.003	0.03
29	DUSP5	0.37	0.013	4	0.012	0.10
36	MYC	0.34	0.017	984	0.002	0.06
37	ROS1	0.34	0.017	84	0.005	0.03
38	HIF1A	0.34	0.017	185	0.007	0.08
42	MIR554	0.34	0.018	0	0.004	0.15
45	IL24	0.32	0.022	0	0.003	0.06
49	TGFB2	0.31	0.025	121	0.008	0.04
51	TGFB1	0.30	0.027	887	0.004	0.03
52	JUNB	0.30	0.028	54	0.008	0.05

### Integral score

The *integral score* is intended to quantify the area between the expression profiles for control and treatment. To compute the integral between the two expression dynamics of each gene *i* we first linearly interpolate the missing values of the quantile regression at measured time points *t* and at time points where the curves intersect. We then estimate the area between the two dynamics D_*i*_ applying the trapezium rule. This integral

$$\mathcal{I}_{i} := \int_{t_{1}}^{t_{2}} \left|\mathrm{D}_{i}^{\text{stim}}(t)-\mathrm{D}_{i}^{\text{ctrl}}(t)\right| \text{dt} $$

for each gene *i* serves as a measure for the difference in the mRNA production between the two conditions. Figure 2 C illustrates the *integral score*, which can be computed for different time intervals. Hereby, four separate scores are computed ($\mathcal {I}_{i}^{\text {early}}, \mathcal {I}_{i}^{\text {intermediate}}, \mathcal {I}_{i}^{\text {late}}, \mathcal {I}_{i}^{\text {complete}}$) to distinguish between the early response, the intermediate response, the late response, and the response over the whole period. The first three scores are defined for subsequent time-intervals, which can be defined by the user. These scores allow to distinguish between slowly and rapidly responding genes, and might also be used to distinguish a secondary response from the direct response to the stimulus. By using a *z*-score transformation and averaging of all three integral scores the *combined integral score* $\mathcal {I}_{i}^{\text {comb}}$ is obtained. The *combined integral score* emphasises the largest changes in gene expression for each period stronger than the more outbalancing *complete integral score* $\mathcal {I}_{i}^{\text {complete}}$.

### Relevance score

By using the R package RISmed [34] we query the PubMed database of publications for records that match both the gene name and the condition. For each gene *i* this yields a number of publications *p*_*i*_. We use a log-transformation to normalise *p*_*i*_ between 0 and 1, and obtain the *relevance score*

$$\mathcal{R}_{i} := {\log}_{p_{\text{max}}}(p_{i}), $$

where *p*_max_:= max*i*(*p*_*i*_). This score indicates whether a gene is already well known to be associated with the condition or potentially a new target.

### Consensus score

The *consensus score* is used for the final ranking of the genes and combines the four scores. By merging the *dynamics score* with the *peak score*, *combined integral score* and *relevance score*, and normalising the result to be between 0 and 1, we obtain

$$\mathcal{C}_{i}:=\frac{\breve{\mathcal{D}}_{i}+ \breve{\mathcal{P}}_{i}+ \breve{\mathcal{I}}_{i}^{\text{comb}}+\breve{\mathcal{R}}_{i}}{4}, $$

whereby score $\mathcal {S}$ is z-transformed $\breve {\mathcal {S}}$ before the average is computed. Figure 2 d shows the z-transformed distributions of the score values. To better centre the relevance score distribution, only non-zero values are considered for the z-transformation.

### Significance

Except for the *peak score* we did not define any significance threshold, yet. For the other scores a significance level can be computed by a one-sided, one-group hypothesis test. The program fits the Cauchy, Gamma, log-normal, logistic, normal, Poisson and Weibull distribution to the empirical distribution of score values using the function fitdistr() provided by the R package MASS [35]. The log-normal distribution is only defined for strictly positive values, however, by shifting the *x*−axis it can be fitted in the negative part as well. The obtained significance threshold is transformed back afterwards. The distribution function providing the best fit of the distribution of score values is automatically selected and plotted. To estimate the significance for a differentially expressed gene we provide the *p*-value as well as the effect size [36]. The effect size of the *peak score* is defined as the distance between the expression dynamics, normalised by the maximum distance possible, i.e. the highest expression value within the data set minus the lowest expression value within the data set. The largest observed expression change in our data set covers 25.9% of the whole detection range and represents the effect size. The same normalisation is used for the *instability score* and also for the *integral score*, where the maximum area is given as the maximal distance multiplied by the time period. In the *consensus score*, a gene is considered to be significant, if it is considered significant in at least two scores.

### Method extension for gene set analysis

**Fig. 3:**
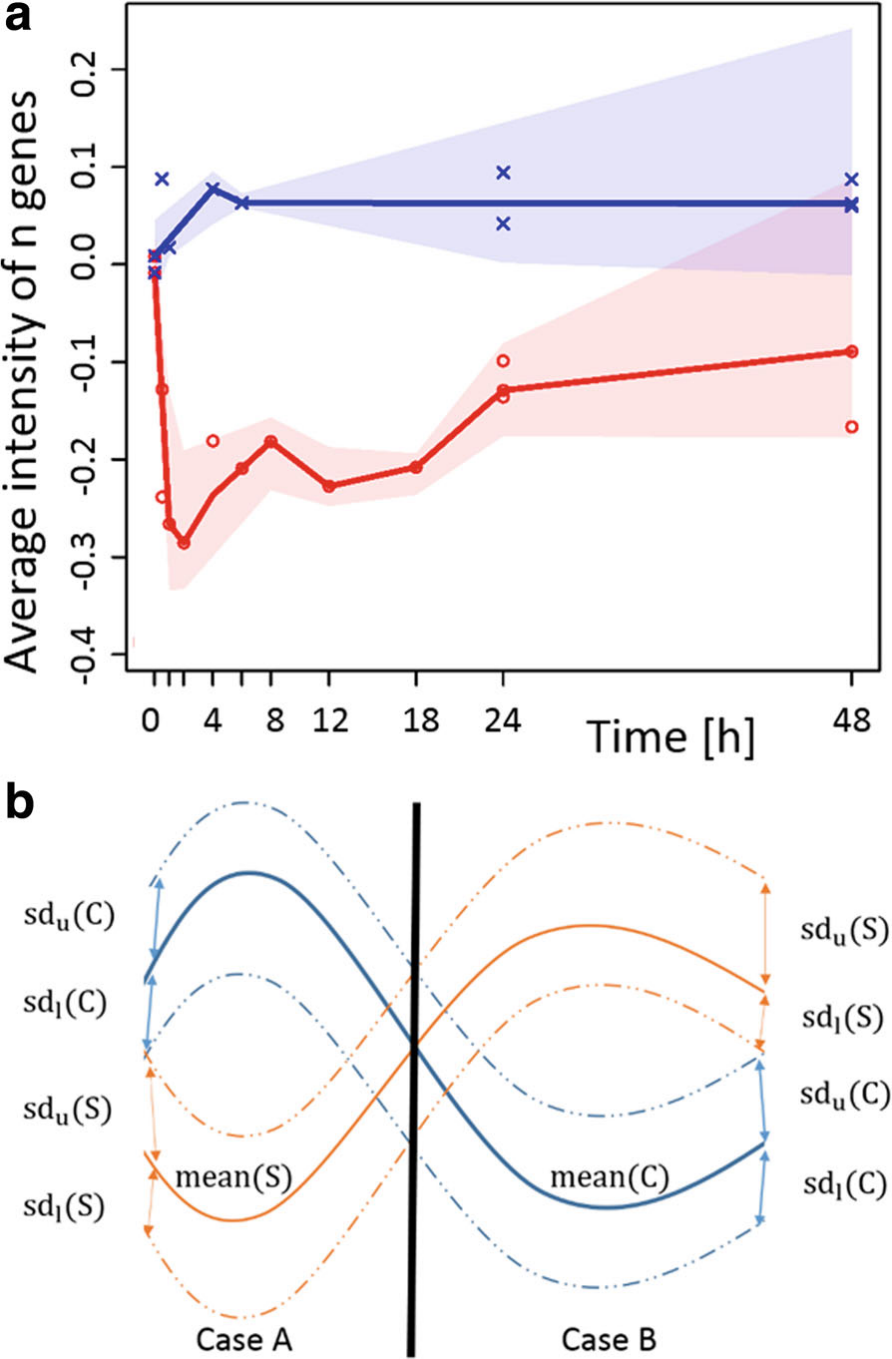
Gene set analysis. **a**) Example analysis result with the average of *n* genes. **b**) Scheme for minimal overlap calculation. The *continuous lines* represent the average expression of the gene group at one time point for either the stimulated sample (S) or the control (C). The *dotted lines* represent the average with *upper* (Sd_*u*_) or *lower* (Sd_*l*_) standard deviation

To investigate the behaviour of functional groups, the genes are linked to gene ontology groups using the BiomaRt-package [37, 38]. Then the expression level at the initial time point is subtracted from the gene expression profile of each gene. Thus, all profiles are initially zero and only the expression change with respect to the first value is observed (Fig. 3 a). Second, the average expression together with the upper *sd*_u_ and lower *sd*_l_ standard deviation of all genes within each ontology group are calculated for each time point. The upper standard deviation hereby accounts for all measurement points above the group mean and the lower standard deviation accounts for all measurement points below the average. Separation into upper and lower standard deviation helps to better recognise when the subset of the functional group shows increased (or decreased) expression. This would lead to enlarged upper (or lower) standard deviations, where the classical standard deviation does not allow such distinction. We then consider gene groups differentially expressed if their expression bandwidths are separated by the condition, i.e., that the variability between genes in the same ontology group are small in contrast to changes caused by different treatments. To test, whether the expression bandwidths are separated by condition, we distinguish two different cases, as shown in Fig. 3 b. On the one hand, the band of the control can be higher than the band of the stimulus (case A), on the other hand, the situation can be reversed (case B). We search for the minimum overlap

$${} \theta_{ij} = \frac{\max \left[ \begin{array}{l} \overbrace{(\text{mean}(\mathrm{C}) - \text{sd}_{\mathrm{l}}(\mathrm{C})) \,-\, (\text{mean}(\mathrm{S}) + \text{sd}_{\mathrm{u}}(\mathrm{S}))}^{\text{Case A}}; \\ \overbrace{(\text{mean}(\mathrm{S}) - \text{sd}_{\mathrm{l}}(\mathrm{S})) \,-\, (\text{mean}(\mathrm{C}) + \text{sd}_{\mathrm{u}}(\mathrm{C}))}^{\text{Case B}} \end{array}\right]}{\underbrace{\frac{1}{2} \left(\text{sd}_{\mathrm{u}} (\mathrm{S}) + \text{sd}_{\mathrm{l}}(S) + \text{sd}_{\mathrm{u}}(\mathrm{C}) + \text{sd}_{\mathrm{l}}(\mathrm{C}) \right)}_{\text{Bandwith}}} $$

of both bandwidths for a combination of time points *j*∈{1,…,*n*} and genes *i*, where *n* indicates the total number of measurements per gene. We are only interested in the maximum distance between the bands or the minimum mutual overlap for the score *Θ*_*i*_= max(*θ*_*i*1_,…, *θ*_*ij*_,…, *θ*_*in*_) at each time point. Positive values indicate a separation of the bands and negative values indicate overlap. The average expression profile for each gene group and treatment is then used to calculate the other scores as described above. Hence, TTCA ranks functional groups high if they contain genes with similar expression pattern over time within a condition and if they clearly change the expression dynamics from one condition to the other. Although we did not compare the performance of the gene set module, the application on real data seems promising. Alternatively, the user can use the ranking of the individual genes to apply other methods for gene set analysis.

### Computation time and further packages

TTCA is computationally fast using about 1 h for one contrast. This includes the analysis of expression dynamics and the generation of relevant figures on a standard laptop (i5 1.70 GHz, memory 12 GB). Furthermore, TTCA uses the R-package tcltk2 [39] for a progress bar and the R-package VennDiagram [40] to show automatically the overlap of significant genes across scores.

## Methods for lung cancer data set

### Cell seeding, growth factor stimulation and microarray processing

The cell line H1975 (NCI-H1975; ATCC: CRL-5908) were obtained from LGC Standards (Teddington, UK). The cell line was authenticated by STR-analysis (DSMZ, Braunschweig, Germany) and routinely checked for mycoplasma contamination. H1975 NSCLC cells were seeded in 6-well-plates with 1.33·10^5^ cells per well. After incubation for 3 days, cells were washed 3 times and supplemented with DMEM without FCS for overnight starvation. On the following day, cells were stimulated with 50 ng/mL of EGF diluted in starvation medium. Samples were harvested after 0, 0.5, 1, 2, 4, 6, 8, 12, 24 and 48 hours. Subsequently RNA was extracted, as described below. Total cellular RNA was isolated with the NucleoSpin RNA II kit according to the manufacturers’ instructions. RNA concentrations were determined by measuring the absorbance (230 - 400 *nm*) using a NanoDrop®;ND-1000 spectrometer. The purity of the RNA was determined through the ratio of the absorbance at 260nm and 280nm. RNA with a ratio ≥ 1.8 was used for further analysis. After assessing RNA integrity using the Agilent Bioanalyzer, 100 *ng* in 3 *μ* *l* per sample were handed over. After amplification, labelling with biotin and fragmentation of the RNA, hybridisation with GeneChip Human Gene 2.0 ST Array was performed for 16 *h* at 45 °C. Subsequently, washing and staining was performed using an Affymetrix Fluidics Station 450 and the microarray was scanned using an Affymetrix GeneArray Scanner 3000.

### Microarray preprocessing

The method Single Channel Array Normalisation (SCAN) [31] was used for the preprocessing. For the mapping of probes to genes we used the Netaffix.v.34 annotation file which is available from the array manufacturer. For the transcript-level we used Brainarray-Ensembl-T-v.18.0.0 [41] for annotation. The quality was additionally assessed before and after preprocessing with the R-package ArrayQualityMetrics [42]. Four possible outliers were visible in the 3D-PCA-plot generated with pcaMethods [43]. They were investigated in contrast to other replicates or to the closest measurement points with Limma [44] and Piano [45] under use of BioMart [46] for GO-mapping. We assumed a problem with the magnesium concentration and excluded the affected arrays from the analysis.

## Results and discussion

**Fig. 4:**
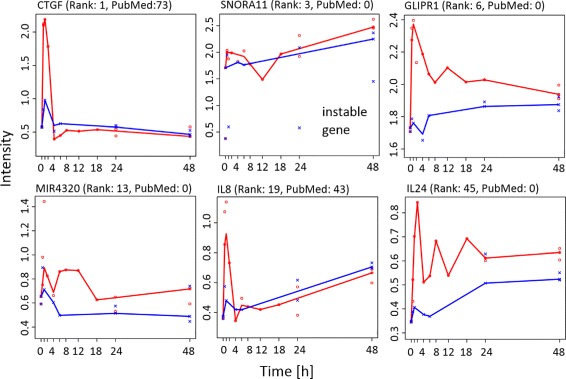
Time course profiles of genes considered significant. *Red*: With EGF stimulation. *Blue*: Control. *Line*: Quantile regression. *Points*: Measurements. SNORA11 is ranked highly significant, but the instability score is high and identifies this finding as false positive

The approach presented here allows the identification of biologically relevant genes from noisy, sparse, and possibly incomplete time course gene expression data sets from perturbation experiments. In the case presented in our study, the administration of the potent mitogen EGF led to the identification of numerous known EGF/EGFR induced target genes as indicated by the *relevance score*, such as CTGF (Fig. 4), EGR1, PTGS2/COX2, and transcription factors of the AP1 family including JUN and FOS (Table 1).

The top-ranked genes represent key factors involved in the initiation and maintenance of a mitogenic response in tumour cells. Interestingly, many of the immediate EGF-dependent targets listed in Table 1 represented transcriptional regulators, for instance EGR1, EGR2, JUN, FOS, or MYC, and secreted chemokines like CTGF, IL8 (Fig. 4), or KITLG/SCF, illustrating that EGF is a central inducer of pro-proliferative gene expression and paracrine regulation in lung cancer. These results are confirmed by previous publications describing for example, that activation of the PI3K/AKT pathway, which typically stimulates the transcription factor AP1 consisting of JUN/FOS heterodimers, can stimulate IL8 production and secretion in NSCLC cells [47].

However, our approach not only confirmed findings from other studies. Even more important, we identified a long list of previously un-published downstream effectors (Additional file 3: Table S4; 18/79 (23%) significantly regulated genes have not been described in the context of EGF/EGFR signalling). For example, the target gene IL24 (Relevance Score: 0.32) has been shown to inhibit NSCLC cell migration suggesting that EGF-induced IL24 might shift tumour cells from a migratory to a mitotic phenotype [48] (Fig. 4). The high ranked gene GLIPR1 (Fig. 4) has recently been identified as tumour suppressor in lung cancer [49], however, the relationship between GLIPR1 and EGF was yet unknown. In addition, the significant regulation of the micro-RNAs miR-4320 (Relevance Score: 0.44; Fig. 4) and miR554 (Relevance Score: 0.34) suggests that EGF supports the oncogenic properties of NSCLC cells via miRNA-dependent mechanisms [50].

We compared TTCA with Limma, EDGE and MaSigPro (see Additional file 2). We assume, that the number of PubMed publications, linking EGF stimulation with individual genes, can be used to generate a ranking of expected target genes. Additional file 2: Table S2 shows the ranking of the top 100 expected genes, determined by TTCA, Limma, EDGE and MaSigPro. Additional file 3: Table S3 shows the top 100 gene names displayed by each method investigated. Additional file 2: Figures S2-S8 show the top ten expression profiles of each method investigated and a *p*-value distribution provided by EDGE. The code for the method comparison is in Additional file 2. The source code of the TTCA method is in Additional file 1.

## Conclusion

We have presented a new method for microarray time-series data analysis, specifically intended for difficult experimental designs with sparse measurements. Even when the experimental design involves a uniform data collection, experimental problems can lead to the exclusion of individual arrays, and thus to the loss of measurement points after quality control. Sufficient replicates are important for proper microarray data analysis [51] and remain important in more accurate next-generation sequencing [52]. However, even if such data are difficult, they nonetheless contain helpful hints for further investigations. TTCA is able to detect different characteristics of the changes in expression dynamics and always provides not only *p*-values but also effect sizes for an optimal significance interpretation [36].

Our method can also be applied for data sets with less complicated designs (regular sampling intervals, large number of replicates) and yield very good results, comparable with other tools. It should be noted, however, that the scores included in TTCA detect specifically expression patterns arising after perturbation or stimulation experiments. For detecting specific dynamical behaviours, e.g. oscillations, we recommend specialised methods like Lomb-Scargle periodograms [53], JTK-CYCLE [54] or GeneCycle [55].

We believe that the developed TTCA package is a valuable and efficient tool for the dissection of important information that is usually concealed by experimental and biological variations leading to data heterogeneity. The connection with the number of PubMed publications has to our knowledge never been included in other packages and supports the user in distinguishing between new and already known genes affected by the applied perturbation. Further new features (at least to our knowledge) are the automatic detection of the best density function, the approach to detect false positives (the instability score), or the distinction between early, middle and late response. Also, the outbalancing of the sampling design using weighting factors is an important new feature. Moreover, we provide a new gene set significance approach, which pools genes into gene ontology groups which expression bandwidths are separated (minimal overlap score). TTCA provides automatically quality checks and plots the gene expression profiles. Thus, the user can easily judge the performance of the package for any included data set. Strong advantages of TTCA are the high degree of transparency, the multitude of visual output for quality assessment, search flexibility and sensitivity also in cases where other methods cannot be applied.

## Additional files

Additional file 1 R code of TTCA (EUPL). (TXT 816 kb)

Additional file 2 Method comparison. A table summarises the methods mentioned in the introduction, method shortcomings are further discussed and TTCA is compared with some applicable methods. Includes **Table S1**-**S3** and **Figures S1**-**S8**. (PDF 2165 kb)

Additional file 3 The complete result table is given in **Table S4**. (XLSX 816 kb)

## Abbreviations

AKT: AKT serine/threonine kinase 1
ANOVA: Analysis of variance
AP1: Activator protein 1
AREG: Amphiregulin
BATS: Bayesian analysis of time series
BETR: Bayesian estimation of temporal regulation
COX2: Cytochrome C oxidase subunit II (see new name: PTGS2)
CRAN: Comprehensive R archive network
CTGF: Connective tissue growth factor
DMEM: Dulbecco’
s modified eagle medium; DUSP5: Dual specificity phosphatase 5
EDGE: Extraction of differential gene expression
EGF: Epidermal growth factor
EGFR: Epidermal growth factor receptor
EGR1: Early growth response 1
EGR2: Early growth response 2
EUPL: European union public licence
F3: Coagulation factor III (thromboplastin, tissue factor)
FCS: Fetal calf serum
FOS: FBJ murine osteosarcoma viral oncogene homolog
FPCA: Functional principle component analysis
FWER: Family wise error rate
GLIPR1: Glioma pathogenesis-related protein 1
GO: Gene ontology
HIF1A: Hypoxia-inducible factor 1-alpha
IL8: Interleukin 8
IL24: Interleukin 24
JUN: Jun proto-oncogene
JUNB: Transcription factor jun-B
KIT: V-Kit Hardy-Zuckerman 4 feline sarcoma viral oncogene homolog
KITLG: KIT ligand
Limma: Linear models for microarray data
MaSigPro: Microarray significant profiles
MIR4320: MicroRNA 4320
MIR554: MicroRNA 554
MYC: V-myc avian myelocytomatosis viral oncogene homolog
NSCLC: Non-small cell lung cancer
PACE: Principle component analysis through conditional expectation
PCA: Principle component analysis
PCNA: Proliferating cell nuclear antigen
PCR: Polymerase chain reaction
PI3K: Phosphatidylinositol 3-kinase
Piano: Platform for integrative analysis of omics data
PTGS2: Prostaglandin-endoperoxide synthase 1
RMA: Robust multi-array average
RNA: Ribonucleic acid
ROS1: Proto-oncogene tyrosine-protein kinase ROS
SAM: Significance analysis of microarrays
SCAN: Single-channel array normalisation
SCF: Stem cell factor (see new name KITLG)
SNORA11: Small nucleolar RNA, H/ACA box 11
TGFB1: Transforming growth factor beta 1
TGFB2: Transforming growth factor beta 2
TTCA: Transcript time course analysis
