# Supplementary material for: TTCA: an R package for the identification of differentially expressed genes in time course microarray data
Source: BMC Bioinformatics. 2017 Jan 14;18:33. doi: 10.1186/s12859-016-1440-8 (PMC5237546; doi:10.1186/s12859-016-1440-8)
Supplement: Additional file 2 — Method comparison. A table summarises the methods mentioned in the introduction, method shortcomings are further discussed and TTCA is compared with some applicable methods. Includes Table S1-S3 and Figures S1-S8. (PDF 2165 kb) [file 12859_2016_1440_MOESM2_ESM.pdf]

TTCA: An R package for the identification of differentially expressed genes in time course microarray data - Supplementary Information 1

Marco Albrecht, Damian Stichel, Benedikt Müller, Ruth Merkle, Carsten Sticht, Norbert Gretz, Ursula Klingmüller, Kai Breuhahn and Franziska Matthäus

Method test: FPCA

We applied the FPCA code on another EGF data set (GSE34228) with dense longitudinal replication. Figure S8 A left shows a good fit of the original data for one highly significant gene. As a control we included the original data by ourselves and did not trust the original data displayed by FPCA. We saw strong differences between our original values and original values displayed by FPCA shown in Figure S1 A right. Also in our data set we observed differences between the fit and our original data as shown in Figure S1 B. We applied FPCA to a further data set of NSCLC cells stimulated with HGF and TGFβ. The PCR measurement of a known target gene (Figure S1 C left) exhibits specific dynamics which are no longer present after applying FPCA (Figure S1 C right). Also the p-value of 0.53 is not what we expected for a target gene after stimulation.

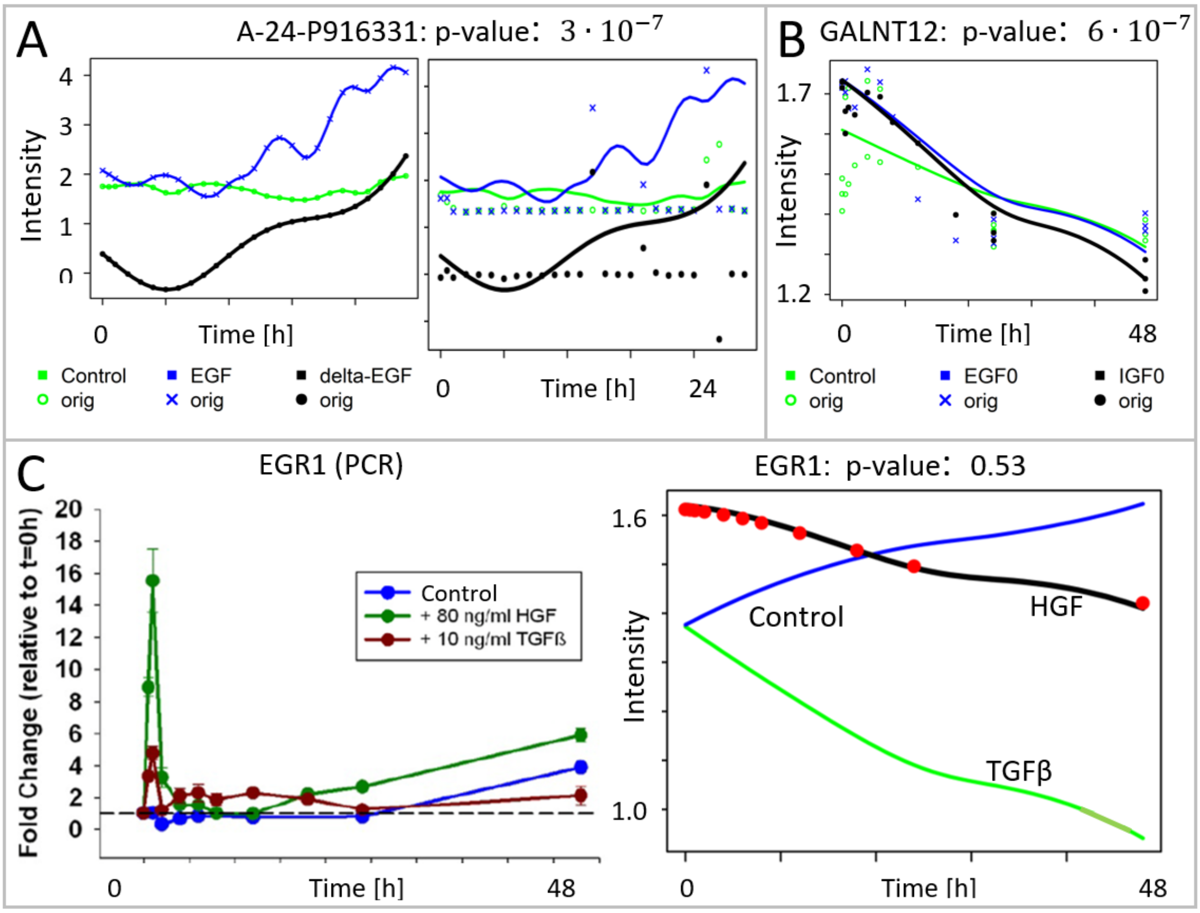

Figure S1: A) FPCA application on data set GSE34228. Left: Seemingly good function fit to the "original" values displayed by FPCA. Right: Unprocessed original data with function fit by FPCA. B) Typical stiff function fit by FPCA with our data set and with our original measurement points. C) Left: Target gene of interest which is measured with PCR. Right: Output of FPCA shows "original" measurement data and function fit. P-value of known target gene is considered as not significant by FPCA.

Method comparison: Overview

Table S1: Method overview. Summarizes methods with comments about their applicability.

METHOD	CITE	PROBLEM
**SAM**	[1]	Only contrast, no time course analysis
**ANOVA**	[2]	Only contrast, no time course analysis
**LIMMA**	[3]	Only contrast, no time course analysis, time course analysis possible using regression spline or a polynomial (not published time course method), our data set was processed in 1.65 seconds.
**EDGE**	[5,6]	Our data set was processed in 22 seconds, however, expected genes were not detected.
**MASIGPRO**	[7]	Expected genes are not detected, provides separate p-values: one for single time course and one for contrast. Detect significant genes for subsequent gene cluster analysis. Our data set was processed in 6.3 minutes.
**SOHN ET AL**	[8,9]	P-value granularity. Expected genes are not detected. Paper describes quantile regression with penalty term, but in practice the simpler function rq() without penalty term is used for quantile regression. Quantil regression with penalty term is implemented in function rqss() in Quantreg package. Method becomes computational too expensive using the correct function.
**R PACKAGE TIMECOURSE**	[10]	No significance threshold. Requires time point replicates.
**BETR**	[11]	Requires time point replicates.
**NETWORK-BASED METHODS**	[12]	Coded in C++ only. Tested with four time point replicates.
**NETWORK-BASED METHODS**	[13]	NACEP is coded in C and can be used in R. Requires time courses with the same number of time points. NACEP clusters genes as first step and the detection of a particular genes depends on other genes. Focus is on gene regulated networks, transcription factors and differentiation processes: http://systemsbio.ucsd.edu/NACEP/
**GAUSSIAN PROCESSES**	[15]	One channel experiments, two sample time course, tested with 4 biological replicates. At least 2 replicates required to build a normally distributed random variable. R-package gprege; Python code http://www.inference.phy.cam.ac.uk/os252/projects/GPTwoSample/
**GAUSSIAN PROCESSES**	[16]	At least 2 replicates required, two channel experiments, one sample time course
**BATS**	[17, 18]	Two channel experiments, one sample time course, link to matlab file does not work. Requires at minimum 5–6 time points.
**METHOD BASED ON PCA**	[19]	No code or software package provided
**METHOD BASED ON FPCA**	[23]	Calculates the dynamics of only one time course. Strong deformation of original time courses, time expensive (>1.5h) and requires the same time-point sampling for both conditions. To compare two time courses one has to subtract one from the other. Applied on data sets for changes in immune system over days. Code not accessible anymore.

**SLIDING WINDOW**	[4]	Requires equidistant time sampling
**MEASURING DISTANCES OR AREAS BETWEEN THE CURVES**	[26, 27]	Useful but problems with gaps or with time courses sampled on different time points. Does not make full use of data.

Method comparison: Top 100 expected genes

We used the number of publications in PubMed that contain the term epidermal growth factor and the gene name of interest as a criterion for expected genes and list in Table S2 the related detection ranking for the methods: TTCA, EDGE, Limma and MaSigPro. Empty fields represent not detected genes.

Table S2: We applied TTCA, EDGE, Limma and MaSigPro to our EGF data set and display the resulting ranking. We searched the number of publications in PubMed that include EGF and the gene name. Genes with the most publications with respective to EGF are ranked high.

PubMed	gene_name	TTCA_rank	EDGE_rank	Limma_rank	MaSigPro_rank	probeset_id
7167	JUN	5	16250			16687875
1437	MET	79	1241			17050591
997	MYC	37	24427			17072669
922	FOS	7	10672			16786587
903	TGFB1	51	3713	105		16872551
885	HBEGF	12	11792			17000724
818	PTGS2	4	2453	86	196	16697370
746	MKI67	22	564	1140		16719515
590	PCNA	21	4410	1343		16916958
569	AREG	8	5126	16	129	16967863
445	GAN	142	15241			16821280
341	CD44	73	6444	1518	146	16723614
283	NRG1	141	29357			17067696
237	BRCA1	92	8087			16845349
230	CEACAM5	71	613			16862548
225	EREG	63	4484	753		16967843
192	HIF1A	38	11595			16785083
191	BDNF	131	2829			16736861
127	ICAM1	103	25776			16858137
124	TGFB2	48	1560	62	392	16677556
121	CCL2	64	25231	1023		16833204
121	CP	119	4491	650		16960304
108	SOX2	86	7338			16948461
106	EGR1	2	14805			16989736
103	ROS1	35	13575		67	17022996
96	CISH	185	7406		410	16954567
93	THBS1	39	7903			16799315
85	KDR	109	5070	1334		16976029
79	MFGE8	154	11766			16813038

76	CTGF	1	11236			17023646
72	F3	16	10002	355		16689869
64	NMB	156	7684	1112	274	16812824
55	JUNB	52	15123			16858710
54	E2F1	58	12543			16918445
53	BMP2	90	11852			16911261
53	ADRB2	65	13949			16990848
50	TOP2A	66	144	426		16844312
48	IL8	19	9793			16967771
47	MAP2K2	183	12383			16867240
45	SOX9	223	22003	1386		16837418
45	TP63	102	485	1280		16949537
45	EBP	114	10044	369		17103327
44	EPHA2	98	12174	1438		16682098
43	LGALS3	173	10906			16784381
40	GRB7	139	3796			16833876
40	DUSP1	11	15020			17002846
39	CDK1	81	3721			16705159
39	HAS2	50	15079			17080648
39	ABO	59	4762	920	198	17099463
38	ILK	229	8285			16721479
38	UBC	53	11630			16758874
38	UBC	243	12587			16772172
33	RAD51	93	10533	777		16799637
32	NCL	267	26216			16909491
28	VGF	214	8998	1590		17060824
27	NOS3	180	1866	15	359	17053455
26	ERRFI1	70	7675	719	145	16681304
26	MCL1	97	12058			16692775
26	PTHLH	286	12188			16762661
24	IL18	220	7106			16744415
24	CD274	17	8474			17083357
23	IGFBP4	343	258	1519		16834091
23	SRF	202	21758			17008856
22	SIRT1	282	11208	866		16705313
22	H2AFX	293	15445			16745236
22	DUSP6	44	4517		397	16768297
21	VCAN	393	11351			16986913
20	ID1	200	5973	435		16912362
20	CDC25A	56	5195			16953279
19	SERPINE1	30	6553	13		17049676
18	LAMC2	77	2791			16674845
17	CLDN1	10	20284	14	250	16962661
17	LIFR	254	4114			16995500
16	CYR61	9	15420			16666738
16	ADORA1	377	3014			16676130

16	NR4A1	78	11734			16751438
16	JAG1	355	8034	693		16917183
15	SRM	124	18977			16681611
15	DKK1	128	19353	870		16705011
15	TYMS	68	184			16850477
15	DNER	291	3733			16909319
15	PDGFA	302	14713			17054243
14	PDCD4	278	1342	1073		16709201
14	FOSB	106	10748			16863287
14	PTGER4	46	12152			16984287
13	ZFP36	84	13967			16861997
13	PTGER1	542	16825			16869639
12	LRP5	158	23536	1313	16	16728066
12	LGALS1	368	10134			16929855
11	PLK1	134	3104			16817017
11	SPRY4	181	15569			17001063
10	SFN	429	14669			16661314
10	RHOG	535	17467			16734744
10	BLM	322	2494			16804902
10	CCNE1	550	17278			16860418
10	VASP	171	23771	93		16863307
10	SNX5	26	4693		390	16917504
10	SPRY1	61	13025	166		16970435
10	LIMK1	682	26975			17047045
10	SNAI2	25	22004			17077004

Method comparison: Top ranked genes

We display in Table S3 the top ranked gene names for the EGF stimulated data set.

Table S3: Method comparison of TTCA, EDGE, Limma and MaSigPro, showing the top 100 ranked genes.

Rank	TTCA	EDGE	LIMMA	MaSigPro
1	CTGF	OTTHUMG00000166293	VTCN1	LINC00685
2	EGR1	TMEM50B	TMEM156	LINC00685
3	SNORA11	CEP55	LOC646862	FMNL2
4	PTGS2	LINC00888	ENTPD3	DNAH2
5	JUN	ENTPD3	ATG9B	HIST2H2BF
6	GLIPR1	MARCH3	TP53INP1	DPP3
7	FOS	OTTHUMG00000015981	ANGPTL4	HMGB3
8	AREG	PCDHB14	HIST2H2BF	PLLP
9	CYR61	HCFC2	TMPRSS11E	DSC2
10	CLDN1	NKX6-1	OGFRL1	CPEB2
11	DUSP1	KIAA1239	ANKRD29	TRIB3

12	HBEGF	TMPRSS11E	OTTHUMG00000159060	ENDOD1
13	TMPRSS11E	IFNG-AS1	SERPINE1	SNORD105B
14	MIR4320	OTTHUMG00000152758	CLDN1	ERV3-1
15	CEACAM6	NUSAP1	NOS3	AIM2
16	F3	ANKRD29	AREG	LRP5
17	CD274	MIR4532	NREP	SLC1A4
18	MCM2	SNX2	MIR205	DESI2
19	IL8	CD53	PLEKHS1	MGAT4A
20	EGR2	COLGALT1	NT5E	CNN3
21	PCNA	LOC440040	ANKRD36	TP53INP1
22	MKI67	ZNF607	DOCK11	KMO
23	EDN1	ATG9B	THSD7A	C19orf82
24	EXO1	NGEF	PTRF	LCP1
25	SNAI2	ERV3-1	ERV3-1	IL1RL1
26	SNX5	DISC2	OTTHUMG00000154884	ICMT
27	ENTPD3	OTTHUMG00000154884	CLCF1	EEF1B2
28	SNORA38B	MICA	OTTHUMG00000172590	NBAS
29	DUSP5	ARHGAP11A	ACO1	FOXM1
30	SERPINE1	OR52K1	MYBL2	CATSPERB
31	TNFAIP3	NTSR2	ARHGEF38	ISOC2
32	GBP2	C15orf60	MIR4742	GSDMB
33	MCM3	C11orf63	GGT8P	ARAP2
34	NR4A3	AURKB	TRMT61A	ORAI3
35	ROS1	TMEM156	CXCL17	ARHGAP5-AS1
36	SCARNA4	OTTHUMG00000002945	C1orf210	TRIM55
37	MYC	BEND3P3	SNORD59B	LMF1
38	HIF1A	TTK	ITGA2	TXNRD1
39	THBS1	DEPDC1	SUOX	ACLY
40	NAPSA	OTTHUMG00000161058	CDKN3	POLA2
41	GBP1	CST11	FYB	SEMA3B
42	NAMPT	ABCA12	STEAP4	RAI1
43	MIR554	SAC3D1	LOC100128822	OTTHUMG00000169663
44	DUSP6	ASPM	UNC13B	PPM1K
45	IL24	PLEKHA6	SNORA64	CTNNBL1
46	PTGER4	PLEK2	CEACAM6	CTSZ
47	OTTHUMG00000171410	OTTHUMG00000178553	SQRDL	HMGCS1
48	TGFB2	MUC5B	SNHG15	ASNS

49	OTTHUMG00000018491	ZNF549	RPSAP52	VCL
50	HAS2	OTTHUMG00000156146	ENO1	HS2ST1
51	TGFB1	FAM9C	AREGB	AQP11
52	JUNB	SLC27A2	SPTLC3	REPIN1
53	UBC	PIK3IP1	PHLDA3	AKTIP
54	MYBL2	AHCYL2	KANK2	NPPA
55	GINS4	LOC399715	OTTHUMG00000168899	HBS1L
56	CDC25A	CD68	OTTHUMG00000169612	ITGA2
57	DHRS3	BATF3	KLHL5	TMEM50B
58	E2F1	OTTHUMG00000018047	HLA-DRA	CHST14
59	ABO	PRDM11	NDUFA5	PPP2R3A
60	ANGPTL4	PXMP2	KLHDC2	JDP2
61	SPRY1	MICB	LOC652993	LOC100506636
62	SEMA7A	MICB	TGFB2	ZNF554
63	EREG	MUC20	KRTAP2-3	DBNDD1
64	CCL2	KLHL6-AS1	TNKS2-AS1	ZNF493
65	ADRB2	FREM2-AS1	ANKRD33	S100A10
66	TOP2A	C17orf53	OTTHUMG00000170592	FLJ39739
67	PDLIM5	KRT17	DOLPP1	ROS1
68	TYMS	SNORA24	TMEM158	ZNF117
69	MT1X	GLUD1P2	FBLL1	LOC731275
70	ERRFI1	RNA5SP442	MIR4461	TEX10
71	CEACAM5	SLC25A5-AS1	SLC11A2	WFDC10B
72	TMEM156	OR7G2	WHAMMP2	LOC100130887
73	CD44	OTTHUMG00000160737	EEPD1	POLM
74	LOC100507507	C3orf27	ZNF493	IGF2R
75	KLF10	FAM178A	PPM1H	TPGS2
76	BLID	MRPL42P5	OSBPL7	SLC11A2
77	LAMC2	HOXD-AS1	MIR548I1	MCCC1
78	NR4A1	MICB	ATP13A5	CDK17
79	MET	MAFG-AS1	FYCO1	KHNYN
80	KLF6	ARHGEF38-IT1	MAZ	CCDC134
81	CDK1	VCY	TTLL11-IT1	AFF1
82	MCM6	VCY	ZNF117	TRMT6
83	CXCL17	PAPSS2	GPR89A	ZNF585A

84	ZFP36	OTTHUMG00000164903	CERS6-AS1	ARL14
85	CPEB2	SNORD59B	IQGAP3	CHAC1
86	SOX2	TLCD1	PTGS2	OSBPL9
87	TK1	POU2F3	PCDHB13	CCSAP
88	SLCO2A1	C9orf106	ACPP	TRIQK
89	AREGB	MUC13	CROT	NAA15
90	BMP2	PNPLA3	GPN1	MPZL2
91	PTRF	BUB1	LOC100128881	ZHX2
92	BRCA1	ACO1	DNAJC14	SLC6A8
93	RAD51	PSG11	VASP	FAM151B
94	RASA4	DKFZp566F0947	FAM50B	CASP2
95	SNORA74A	FHL1	EEF1G	SLC2A12
96	CDC20	LOC100506257	ADHFE1	UHRF2
97	MCL1	OTTHUMG00000158558	G0S2	LSS
98	EPHA2	OR7E47P	OTTHUMG00000164709	MPZL3
99	KRTAP2-3	LOC100506393	PATZ1	PPP1R13B
100	NAB2	LOC100507003	FAM27E2	MIR205

Method test: EDGE

EDGE delivered no results with the given experimental design using the most recent method: “optimal discovery procedure”. We excluded measurement points that were only sampled for one condition but not for the other, and we averaged the available replicates. We applied the bootstrap approach. The top 10 ranking is shown in Figure S2 and S3. EDGE has problems with the data set, as shown in the conservative p-value distribution in Figure S 4, automatically produced by EDGE. The best ranked gene is background noise as measurement values are negative. SCAN pre-processing subtract background noise from original values. EDGE prefers genes with low expression levels, because the variance is then very low as well. In contrast, TTCA removes negative values and takes the effect size into account.

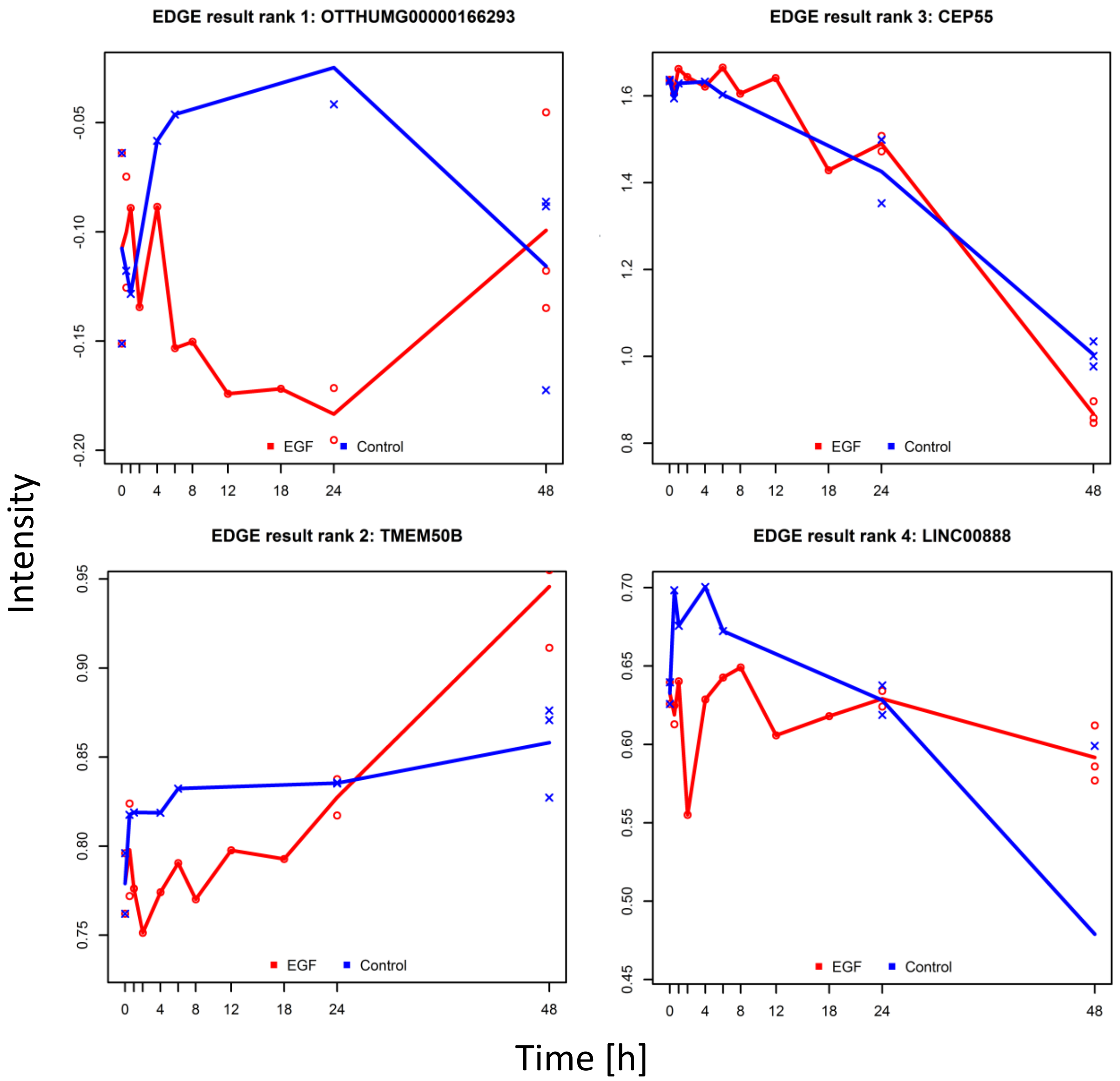

Figure S2: EDGE ranking 1 to 4. Shows gene expression profiles of top selected genes. Points represent measurement data. Solid lines are step wise interpolations of measurement points only for graphical purposes. Red: Stimulation with EGF. Blue: Control.

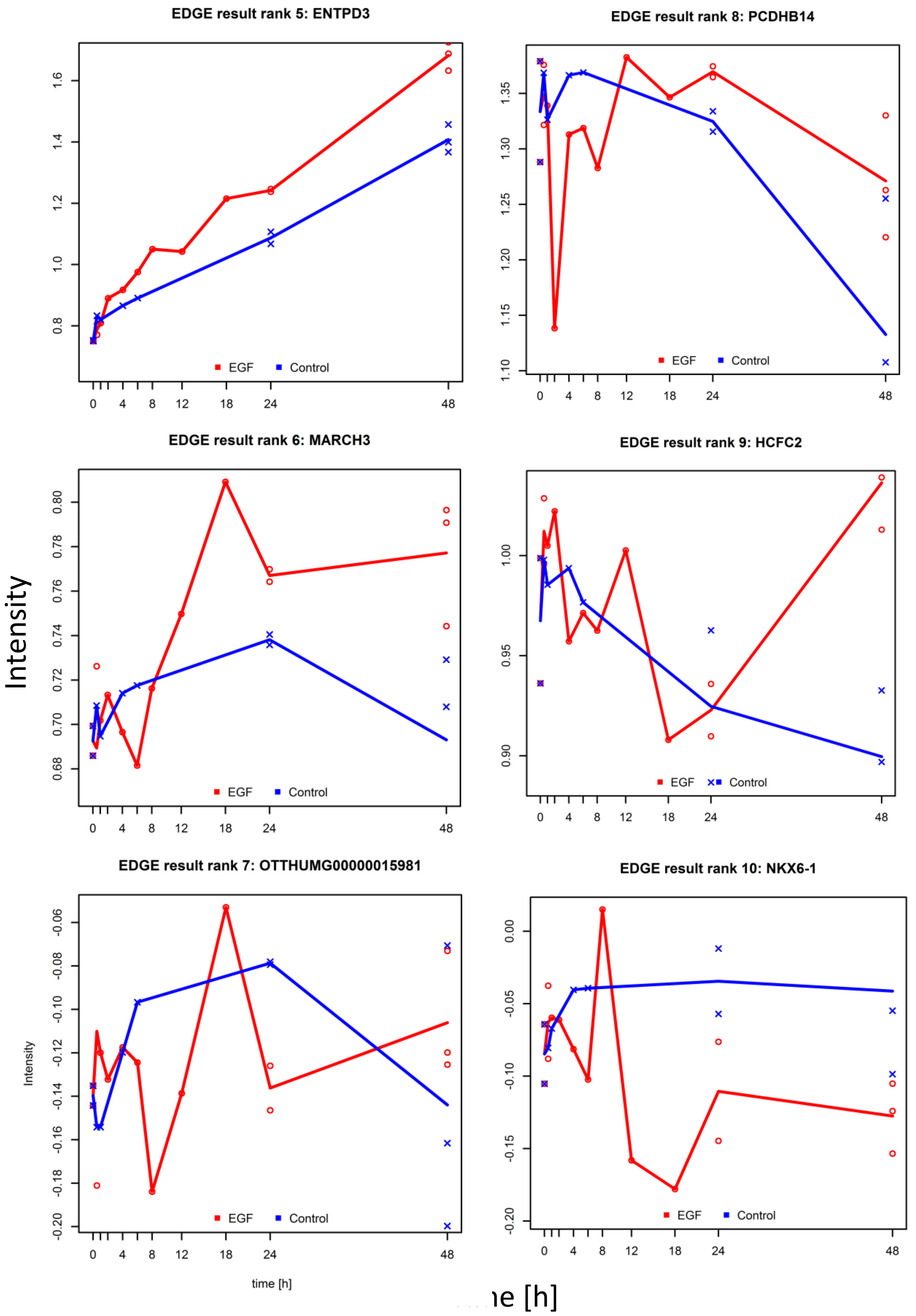

Figure S3: EDGE ranking 5 to 10. Shows gene expression profiles of top selected genes. Points represent measurement data. Solid lines are step wise interpolations of measurement points only for graphical purposes. Red: Stimulation with EGF. Blue: Control.

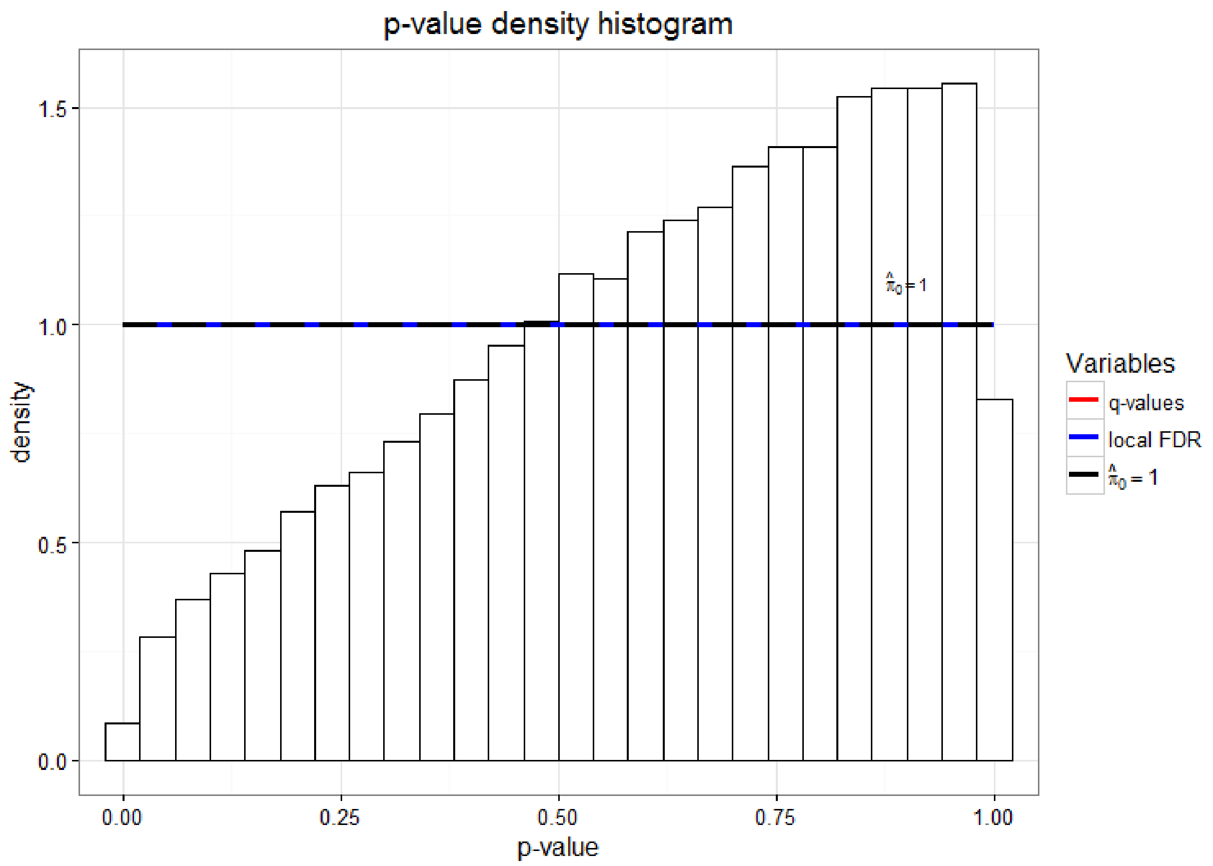

Figure S4: Quality plot provided by EDGE. Red: Below black solid line, all q-values are 1. Conservative p-value distribution

Method test: Limma

We applied Limma to our data set shown in Figure S5 and S6.

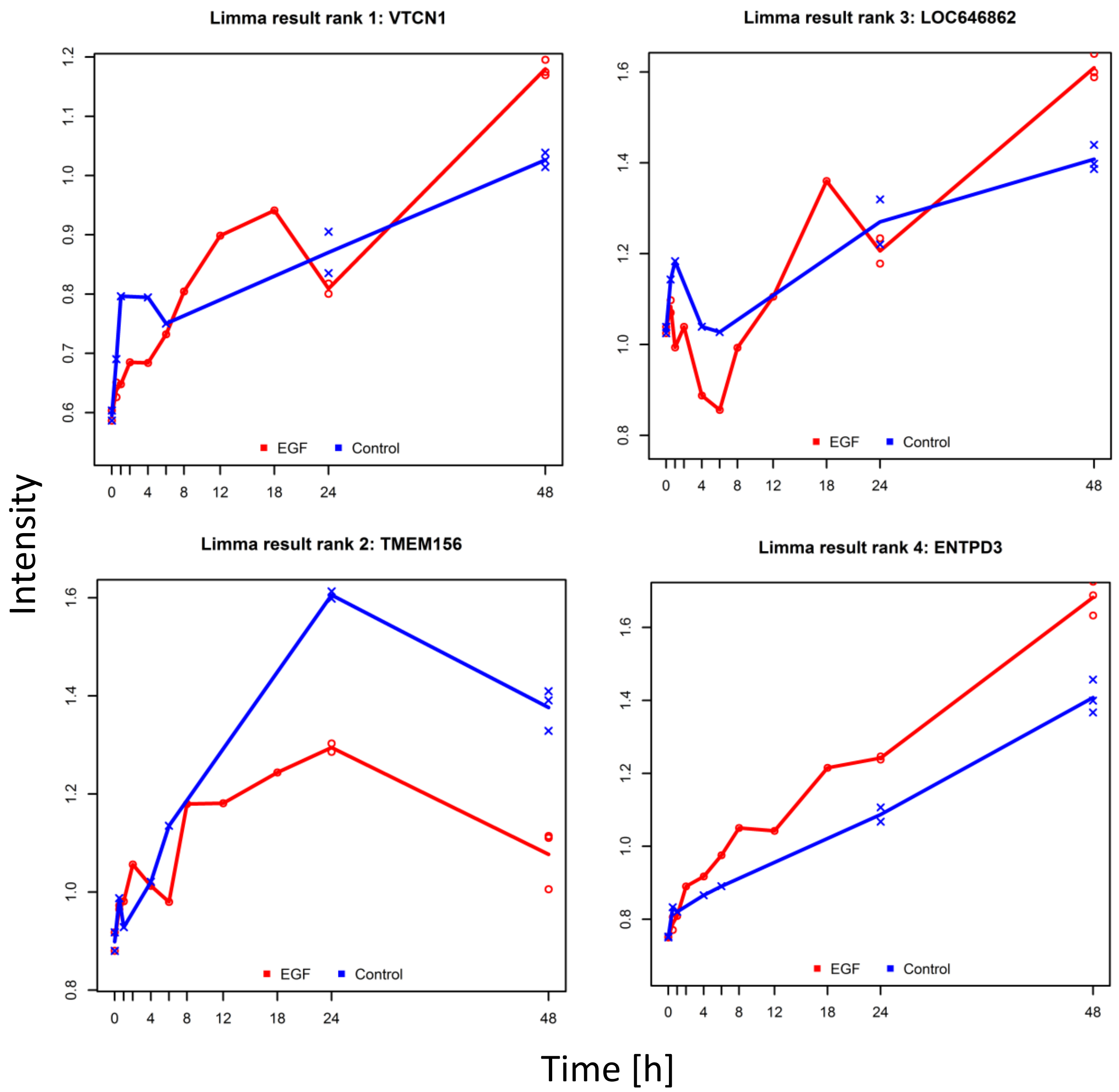

Figure S5: Limma ranking 1 to 4: Shows gene expression profiles of top selected genes. Points represent measurement data. Solid lines are step wise interpolations of measurement points only for graphical purposes. Red: Stimulation with EGF. Blue: Control.

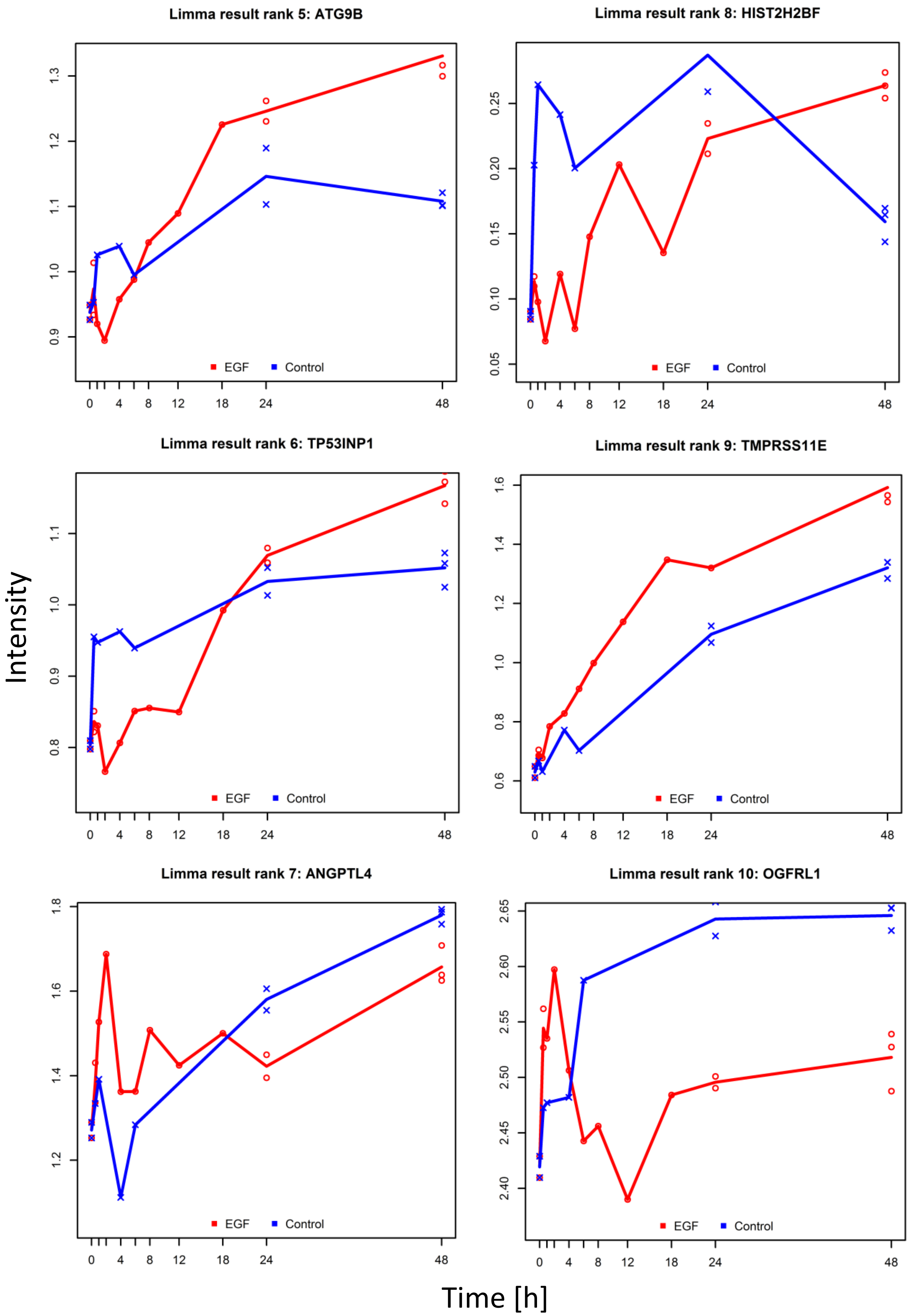

Figure S6: Limma ranking 5 to 10: Shows gene expression profiles of top selected genes. Points represent measurement data. Solid lines are step wise interpolations of measurement points only for graphical purposes. Red: Stimulation with EGF. Blue: Control.

Method test: MaSigPro

We applied MaSigPro to our data set shown in Figure S7 and S8.

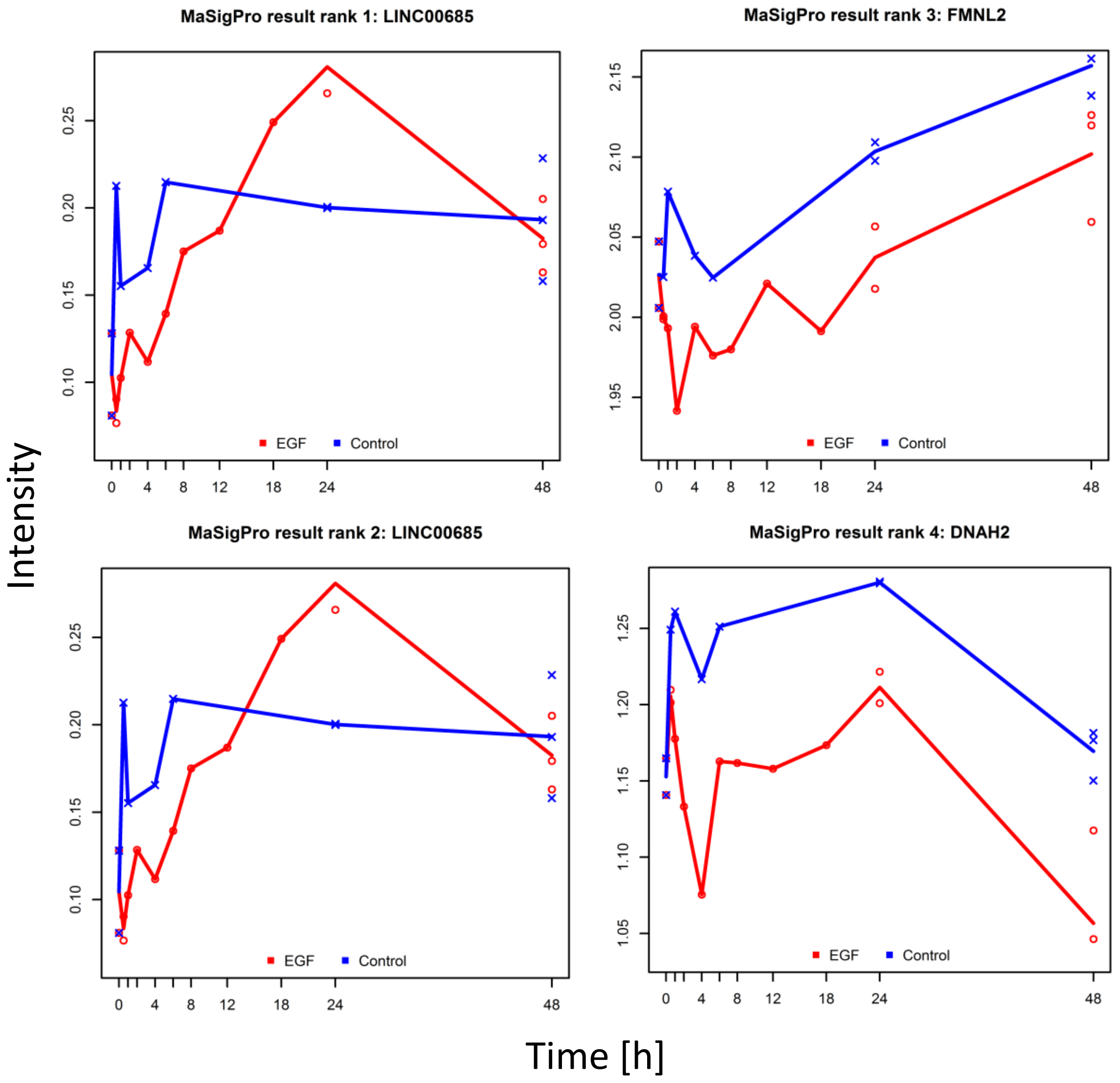

Figure S7: MaSigPro ranking 1 to 4: Shows gene expression profiles of top selected genes. Points represent measurement data. Solid lines are step wise interpolations of measurement points only for graphical purposes. Red: Stimulation with EGF. Blue: Control

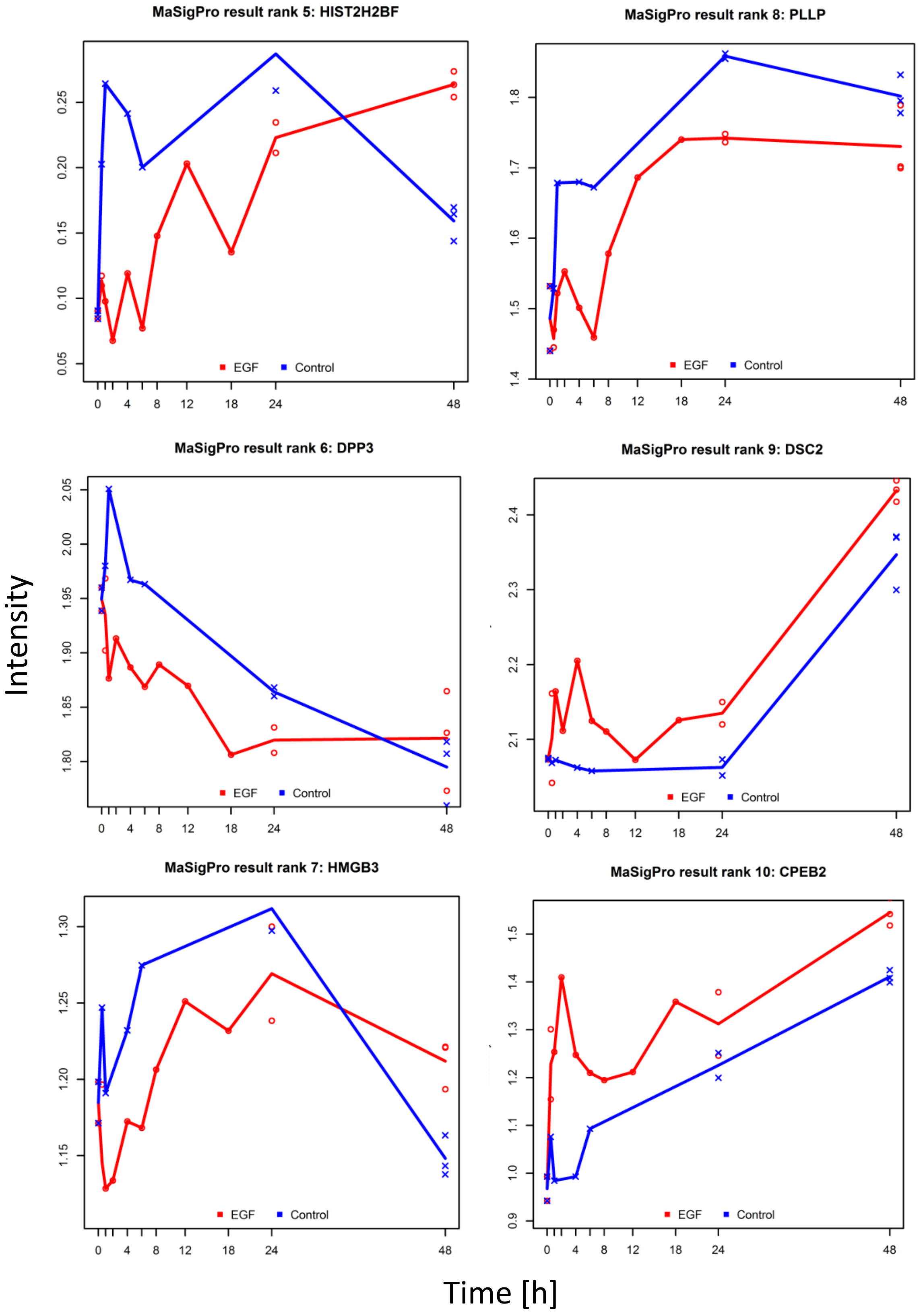

Figure S8: MaSigPro ranking 5 to 10: Shows gene expression profiles of top selected genes. Points represent measurement data. Solid lines are step wise interpolations of measurement points only for graphical purposes. Red: Stimulation with EGF. Blue: Control

R-Code

```
##########################################
#### Gene-analysis
load("C:/Users/Marco
Albrecht/Documents/CompBioSys/Affymetrix/PreprocessAndQC/Breuhahnexpression_SCAN_NetaffixTranscr_34.RData")
rownames(resultB)<-resultB[,1]
######
load("C:/Users/Marco Albrecht/Documents/CompBioSys/Affymetrix/PreprocessAndQC/Annotation_Transkript_changed_Netaff.RData")
annotation<-annotation[,c("probeset_id", "gene_name","transkript_id","GO_BP","GO_CC","GO_mf")]
rownames(annotation)<-annotation[,1]
annotation<-annotation[,-1]
annot     <-  resultB[,c("probeset_id","gene_name")]
######
EGF<-resultB[ ,
c("B_C_2_0","B_C_3_0","B_EGF_1_0.5","B_EGF_3_0.5","B_EGF_1_1","B_EGF_3_2","B_EGF_1_4","B_EGF_1_6","B_EGF_1_8","B_EGF_2_1
2","B_EGF_2_18","B_EGF_1_24","B_EGF_3_24","B_EGF_1_48","B_EGF_2_48","B_EGF_3_48")]
EGF.time <-  c(0,0,0.5,0.5,1,2,4,6,8,12,18,24,24,48,48,48)
BControl<-resultB[ ,
c("B_C_2_0","B_C_3_0","B_C_2_0.5","B_C_1_1","B_C_3_4","B_C_2_6","B_C_1_24","B_C_3_24","B_C_1_48","B_C_2_48","B_C_3_48")]
BControl.time <-  c(0,0,0.5,1,4,6,24,24,48,48,48)
########


####################################################################################################################
##########################
### TTCA
####################################################################################################################
#############

install.packages("tcltk2")
install.packages("TTCA")
library("TTCA")
TTCA(grp1=EGF, grp1.time=EGF.time, grp2=BControl , grp2.time=BControl.time, lambda = 0.6, annot = annot, annotation = "annotation",
timeInt = c(4,12), pVal = 0.05, codetest = FALSE, file = "C:/Users/Marco Albrecht/Desktop/Pup_Master_MA_V3/method comparison",
MaxPics = 10000, Stimulus1 = "epidermal+growth+factor",
    Stimulus2 = "", S = "gene", mapGO = "", PeakMode = "norm")

####################################################################################################################
##########################
### Limma
####################################################################################################################
#############
ptmL <- proc.time()
#Create an expression set
library("Biobase")
colnames(EGF)[1:2]<- c("B_EGF_2_0", "B_EGF_3_0")
Bind<-cbind(EGF,BControl)
exprs <- as.matrix(Bind)
eset <- ExpressionSet(assayData=exprs)

##############################################################################
#source("https://bioconductor.org/biocLite.R")
#biocLite("limma")
library("limma")
library("splines")
X     <-     ns(c(EGF.time, BControl.time),df=5)
Group <- factor(c(rep("EGF", times = length(EGF.time)), rep("Ctrl", times = length(BControl.time))))
design <- model.matrix(~Group*X)
fit <- lmFit(eset, design)
fit <- eBayes(fit)
TT<-topTable(fit, coef=8:12, number=500000)
TT<-TT[TT$P.Value<0.05,]
Result<-merge(TT,annot,by="row.names",all.x=TRUE)
ResultFin<-Result[,c("gene_name", "P.Value", "adj.P.Val","probeset_id")]
ResultFin<-ResultFin[order(ResultFin$P.Value),]
```

```
ResultFin<-ResultFin[complete.cases(ResultFin),]
head(ResultFin)
LimmaTimeConsum<-proc.time() - ptmL
LimmaTimeConsum

for(i in 1:10){
y11<-as.numeric(EGF[ rownames(EGF)==ResultFin$probeset_id[i], ])
x11<-EGF.time
y22<-as.numeric(BControl[ rownames(BControl)==ResultFin$probeset_id[i], ])
x22<-BControl.time

x1<-approx(x11, y11, xout = unique(x11), method = "constant")$x
y1<-approx(x11, y11, xout = unique(x11), method = "constant")$y
x2<-approx(x22, y22, xout = unique(x22), method = "constant")$x
y2<-approx(x22, y22, xout = unique(x22), method = "constant")$y
## Plot
png(filename = paste0("C:/Users/Marco Albrecht/Desktop/Pup_Master_MA_V3/method
comparison/LimmaRank_",as.character(i),".png"),width = 3.25, height    = 3.25,units= "in",res = 1200, pointsize = 6)
plot(x1, y1, ylim=c(min(c(y1,y2))-(0.1*(max(c(y1,y2))-min(c(y1,y2)))),max(c(y1,y2),na.rm = TRUE)),type="l",
col="red",lwd=2,ylab="Intensity", xlab=" time [h]", xaxt="n")
points(x11,y11,col="red")
lines(x2, y2,col="blue",lwd=2)
points(x22,y22 ,col="blue",pch=4)
axis(side=1, at=round(unique(x1,y1)))
title(main = list(paste0("Limma result rank ",as.character(i),": ",ResultFin$gene_name[i]), cex = 1.2))
legend('bottom','groups',c("EGF", "Control"), pch=c(15,15),col=c('red',"blue"),ncol=2,bty ="n")
dev.off()
}
## end Plot

write.table(ResultFin, "C:/Users/Marco Albrecht/Desktop/Pup_Master_MA_V3/method comparison/RESULT_Limma.txt", sep="\t")
rm(ResultFin)


#############################################################################################################
##########################
### EDGE
#############################################################################################################
#############
ptmE <- proc.time()

#source("https://bioconductor.org/biocLite.R")
#biocLite("edge")
library("edge")
library("splines")

# data(endotoxin)
# endoexpr <- endotoxin$endoexpr
# class <- endotoxin$class
# ind <- endotoxin$ind
# time <- endotoxin$time

#because the method doesn´t work with gaps and unbalanced replicated data we removed unmatched arrays and averaged the replicates
EGFred<-cbind(as.data.frame(rowMeans(resultB[,c("B_C_2_0","B_C_3_0")], na.rm = TRUE)),
     as.data.frame(rowMeans(resultB[,c("B_EGF_1_0.5","B_EGF_3_0.5")], na.rm = TRUE)),
     resultB[,c("B_EGF_1_1","B_EGF_1_4","B_EGF_1_6")],
     as.data.frame(rowMeans(resultB[,c("B_EGF_1_24","B_EGF_3_24")], na.rm = TRUE)),
     as.data.frame(rowMeans(resultB[,c("B_EGF_1_48","B_EGF_2_48","B_EGF_3_48")], na.rm = TRUE))
     )
Controlred<-cbind(as.data.frame(rowMeans(resultB[,c("B_C_2_0","B_C_3_0")], na.rm = TRUE)),
           resultB[,c("B_C_2_0.5","B_C_1_1","B_C_3_4","B_C_2_6")],
            as.data.frame(rowMeans(resultB[,c("B_C_1_24","B_C_3_24")], na.rm = TRUE)),
            as.data.frame(rowMeans(resultB[,c("B_C_1_48","B_C_2_48","B_C_3_48")], na.rm = TRUE))
)
Bind<-cbind(EGFred,Controlred)
endoexpr<- as.matrix(Bind)
```

```
colnames(endoexpr) <- NULL
rownames(endoexpr) <- NULL

timeC=c(0,0.5,1,4,6,24,48)
ind<-c(rep(1, times = length(timeC)),rep(2, times = length(timeC)) )
time <-c(timeC,timeC)
class <- factor(c(rep("EGF", times = length(timeC)), rep("Ctrl", times = length(timeC))))


# colnames(EGF)[1:2]<- c("B_EGF_2_0", "B_EGF_3_0")
# Bind<-cbind(EGF,BControl)
# endoexpr<- as.matrix(Bind)
# colnames(endoexpr) <- NULL
# rownames(endoexpr) <- NULL
# head(endoexpr)
# ind<-c(1,2,1,2,1,1,1,1,1,1,1,2,1,2,3,3,4,3,2,2,2,3,4,4,5,6)
# time <-c(EGF.time,BControl.time)
# class <- factor(c(rep("EGF", times = length(EGF.time)), rep("Ctrl", times = length(BControl.time))))
## tried to order the columns to follow the design of the example
# ORD<-order(ind)
# ind<-ind[ORD]
# endoexpr<-endoexpr[,ORD]
# time<-time[ORD]
# class<-class[ORD]

## Possibility 1
#cov <- data.frame(ind = ind, tme = time, grp = class)
#null_model <- ~grp + ns(tme, df = 2, intercept = FALSE)
#null_model <- ~grp + ns(tme, df = 2, intercept = FALSE) + (grp):ns(tme, df = 2, intercept = FALSE)
#de_obj <- build_models(data = endoexpr, cov = cov, full.model = null_model, null.model = null_model)
## Possibility 2
de_obj <- build_study(data = endoexpr, grp = class,  tme = time, ind = ind, sampling = "timecourse")

slotNames(de_obj)
gibexpr <- exprs(de_obj)
head(gibexpr)
cov <- pData(de_obj)
cov

ef_obj <- fit_models(de_obj, stat.type = "lrt")

head(betaCoef(ef_obj))

alt_res <- resFull(ef_obj)
head(alt_res)
null_res <- resNull(ef_obj)
head(null_res)

alt_fitted <- fitFull(ef_obj)
head(alt_fitted)
null_fitted <- fitNull(ef_obj)
head(null_fitted)


#de_lrt <- lrt(de_obj, nullDistn = "normal")
de_lrt <- lrt(de_obj, nullDistn = "bootstrap")
#### "Error with optimal discovery procedure" leads to error
#de_odp <- odp(de_obj, bs.its = 50, verbose = TRUE,  n.mods = 50)


summary(de_lrt)

sig_results <- qvalueObj(de_lrt)
names(sig_results)

hist(sig_results)
```

```
pvalues <- sig_results$pvalues
qvalues <- sig_results$qvalues
lfdr <- sig_results$lfdr
pi0 <- sig_results$pi0

qvalues[2]

fdr.level <- 0.1
sigGenes <- qvalues < fdr.level


EDGEres<-as.data.frame(cbind(pvalues,qvalues))
rownames(EDGEres)<-rownames(EGF)


Result<-merge(EDGEres,annot,by="row.names",all.x=TRUE)
ResultFin<-Result[,c("gene_name", "pvalues", "qvalues","probeset_id")]
ResultFin<-ResultFin[order(ResultFin$pvalues),]
ResultFin<-ResultFin[complete.cases(ResultFin),]
head(ResultFin)
EDGETimeConsum<-proc.time() - ptmE
EDGETimeConsum
for(i in 1:10){
  y11<-as.numeric(EGF[ rownames(EGF)==ResultFin$probeset_id[i], ])
  x11<-EGF.time
  y22<-as.numeric(BControl[ rownames(BControl)==ResultFin$probeset_id[i], ])
  x22<-BControl.time

  x1<-approx(x11, y11, xout = unique(x11), method = "constant")$x
  y1<-approx(x11, y11, xout = unique(x11), method = "constant")$y

  x2<-approx(x22, y22, xout = unique(x22), method = "constant")$x
  y2<-approx(x22, y22, xout = unique(x22), method = "constant")$y
  ## Plot
  png(filename = paste0("C:/Users/Marco Albrecht/Desktop/Pup_Master_MA_V3/method
comparison/EDGERank_",as.character(i),".png"),width = 3.25, height    = 3.25,units= "in",res = 1200, pointsize = 6)
  plot(x1, y1, ylim=c(min(c(y1,y2))-(0.1*(max(c(y1,y2))-min(c(y1,y2)))),max(c(y1,y2),na.rm = TRUE)),type="l",
col="red",lwd=2,ylab="Intensity", xlab=" time [h]", xaxt="n")
  points(x11,y11,col="red")
  lines(x2, y2,col="blue",lwd=2)
  points(x22,y22 ,col="blue",pch=4)
  axis(side=1, at=round(unique(x1,y1)))
  title(main = list(paste0("EDGE result rank ",as.character(i),": ",ResultFin$gene_name[i]), cex = 1.2))
  legend('bottom','groups',c("EGF", "Control"), pch=c(15,15),col=c('red',"blue"),ncol=2,bty ="n")
  dev.off()
}


write.table(ResultFin, "C:/Users/Marco Albrecht/Desktop/Pup_Master_MA_V3/method comparison/RESULT_EDGE.txt", sep="\t")
rm(ResultFin)
#########################################################################################################
##########################
### MaSigPro
#########################################################################################################
#############
ptmM <- proc.time()

library("Biobase")
colnames(EGF)[1:2]<- c("B_EGF_2_0", "B_EGF_3_0")
Bind<-cbind(BControl,EGF)
exprs <- as.matrix(Bind)


colnames(EGF)[1:2]<- c("B_EGF_2_0", "B_EGF_3_0")
M<-as.data.frame(cbind(c(BControl.time,EGF.time),
```

```
                    c(1,1,2,3,4,5,6,6,7,7,7,8,8,9,9,10,11,12,13,14,15,16,17,17,18,18,18),
                    c(rep(1, 11), rep(0, 16) ),
                    c(rep(0, 11), rep(1, 16) )) )
colnames(M)<-c("Time", "Replicate", "control", "EGF")
rownames(M)<-(c(colnames(BControl),colnames(EGF)))


#source("https://bioconductor.org/biocLite.R")
#biocLite("maSigPro")
library("maSigPro")
#maSigProUsersGuide()

design <- make.design.matrix(M, degree = 2)

design$groups.vector


fit <- p.vector(exprs, design, Q = 0.05, MT.adjust = "BH", min.obs = 20)


fit$i # returns the number of significant genes
fit$alfa # gives p-value at the Q false discovery control level
fit$SELEC # is a matrix with the significant genes and their expression values


tstep <- T.fit(fit, step.method = "backward", alfa = 0.05)

sigs <- get.siggenes(tstep, rsq = 0.6, vars = "groups")


N<-sigs$sig.genes$EGFvscontrol$sig.pvalues
head(N)

Result<-merge(N[, c("p-value","p.valor_EGFvscontrol")],annot,by="row.names",all.x=TRUE)
ResultFin<-Result[,c("gene_name", "p-value", "p.valor_EGFvscontrol","probeset_id")]
ResultFin<-ResultFin[order(ResultFin$p.valor_EGFvscontrol),]
ResultFin<-ResultFin[complete.cases(ResultFin),]
head(ResultFin)

MaSigProTimeConsum<-proc.time() - ptmM
MaSigProTimeConsum
for(i in 1:10){
  y11<-as.numeric(EGF[ rownames(EGF)==ResultFin$probeset_id[i], ])
  x11<-EGF.time
  y22<-as.numeric(BControl[ rownames(BControl)==ResultFin$probeset_id[i], ])
  x22<-BControl.time

  x1<-approx(x11, y11, xout = unique(x11), method = "constant")$x
  y1<-approx(x11, y11, xout = unique(x11), method = "constant")$y

  x2<-approx(x22, y22, xout = unique(x22), method = "constant")$x
  y2<-approx(x22, y22, xout = unique(x22), method = "constant")$y
  ## Plot
  png(filename = paste0("C:/Users/Marco Albrecht/Desktop/Pup_Master_MA_V3/method
comparison/MaSigPro_EGFvsCon_Rank_",as.character(i),".png"),width = 3.25, height    = 3.25,units= "in",res = 1200, pointsize = 6)
  plot(x1, y1, ylim=c(min(c(y1,y2))-(0.1*(max(c(y1,y2))-min(c(y1,y2)))),max(c(y1,y2),na.rm = TRUE)),type="l",
col="red",lwd=2,ylab="Intensity", xlab=" time [h]", xaxt="n")
  points(x11,y11,col="red")
  lines(x2, y2,col="blue",lwd=2)
  points(x22,y22 ,col="blue",pch=4)
  axis(side=1, at=round(unique(x1,y1)))
  title(main = list(paste0("Limma result rank ",as.character(i),": ",ResultFin$gene_name[i]), cex = 1.2))
  legend('bottom','groups',c("EGF", "Control"), pch=c(15,15),col=c('red',"blue"),ncol=2,bty ="n")
  dev.off()
}
```

```
write.table(ResultFin, "C:/Users/Marco Albrecht/Desktop/Pup_Master_MA_V3/method comparison/RESULT_MaSigPro_EGFvsCon.txt",
sep="\t")

Result<-merge(N[, c("p-value","p.valor_EGFvscontrol")],annot,by="row.names",all.x=TRUE)
ResultFin<-Result[,c("gene_name", "p-value", "p.valor_EGFvscontrol","probeset_id")]
ResultFin<-ResultFin[order(ResultFin$p-value),]
ResultFin<-ResultFin[complete.cases(ResultFin),]
head(ResultFin)

for(i in 1:10){
  y11<-as.numeric(EGF[ rownames(EGF)==ResultFin$probeset_id[i], ])
  x11<-EGF.time
  y22<-as.numeric(BControl[ rownames(BControl)==ResultFin$probeset_id[i], ])
  x22<-BControl.time

  x1<-approx(x11, y11, xout = unique(x11), method = "constant")$x
  y1<-approx(x11, y11, xout = unique(x11), method = "constant")$y

  x2<-approx(x22, y22, xout = unique(x22), method = "constant")$x
  y2<-approx(x22, y22, xout = unique(x22), method = "constant")$y
  ## Plot
  png(filename = paste0("C:/Users/Marco Albrecht/Desktop/Pup_Master_MA_V3/method
comparison/MaSigPro_pval_Rank_",as.character(i),".png"),width = 3.25, height    = 3.25,units= "in",res = 1200, pointsize = 6)
  plot(x1, y1, ylim=c(min(c(y1,y2))-(0.1*(max(c(y1,y2))-min(c(y1,y2)))),max(c(y1,y2),na.rm = TRUE)),type="l",
col="red",lwd=2,ylab="Intensity", xlab=" time [h]", xaxt="n")
  points(x11,y11,col="red")
  lines(x2, y2,col="blue",lwd=2)
  points(x22,y22 ,col="blue",pch=4)
  axis(side=1, at=round(unique(x1,y1)))
  title(main = list(paste0("MaSigPro result rank ",as.character(i),": ",ResultFin$gene_name[i]), cex = 1.2))
  legend('bottom','groups',c("EGF", "Control"), pch=c(15,15),col=c('red',"blue"),ncol=2,bty ="n")
  dev.off()
}
write.table(ResultFin, "C:/Users/Marco Albrecht/Desktop/Pup_Master_MA_V3/method comparison/RESULT_MaSigPro_pval.txt",
sep="\t")

rm(ResultFin)

##################################################################################################################
##########################
### Data merging
##################################################################################################################
#############

EDGEdata <- read.table("C:/Users/Marco Albrecht/Desktop/Pup_Master_MA_V3/method comparison/RESULT_EDGE.txt", header=TRUE)
EDGEdata <- EDGEdata[order(EDGEdata$pvalues),]
EDGEdata[,5]<-1:length(EDGEdata$pvalues)
colnames(EDGEdata) <- c("gene_name",  "EDGE_pvalues",  "EDGE_qvalues", "probeset_id","EDGE_rank")

Limmadata <- read.table("C:/Users/Marco Albrecht/Desktop/Pup_Master_MA_V3/method comparison/RESULT_Limma.txt",
header=TRUE)
Limmadata <- Limmadata[order(Limmadata$P.Value),]
Limmadata[,5]<-1:length(Limmadata$P.Value)
colnames(Limmadata) <- c("gene_name",  "Limma_pvalues",  "Limma_qvalues", "probeset_id","Limma_rank")

MaSigProdata <- read.table("C:/Users/Marco Albrecht/Desktop/Pup_Master_MA_V3/method
comparison/RESULT_MaSigPro_EGFvsCon.txt", header=TRUE)
MaSigProdata <- MaSigProdata[order(MaSigProdata$p.valor_EGFvscontrol),]
MaSigProdata[,5]<-1:length(MaSigProdata$p.valor_EGFvscontrol)
colnames(MaSigProdata) <- c("gene_name",  "MaSigPro_pvalues",  "MaSigPro_pvalues_EGFvscontrol", "probeset_id","MaSigPro_rank")

TTCAdata     <- read.table("C:/Users/Marco Albrecht/Desktop/Pup_Master_MA_V3/method comparison/RESULT_EGF-vs-
BControl_Pval.tsv", header=TRUE, sep="\t")
TTCAdata     <- TTCAdata[ ,c("gene_name" , "ConsensusScore" , "Pval_ConsensusScore" , "PubMed" , "probeset_id")]
TTCAdata[,6] <- 1:length(TTCAdata$Pval_ConsensusScore)
colnames(TTCAdata) <- c("gene_name",  "TTCA_ConsensusScore",  "TTCA_Pval_ConsensusScore", "PubMed","probeset_id","TTCA_rank")
```

```
M1<-merge(EDGEdata,Limmadata, by = "probeset_id"  , all = TRUE)

M2<-merge(MaSigProdata,TTCAdata, by = "probeset_id"  , all = TRUE)

Result<-merge(M1,M2, by = "probeset_id"  , all = TRUE)


Result <- Result[order(-Result$PubMed),]
head(Result)

write.table(Result, "C:/Users/Marco Albrecht/Desktop/Pup_Master_MA_V3/method comparison/RESULT_AllMethods.tsv", sep="\t")
```
